# A Genome-Wide CRISPR Interference Screen Reveals an StkP-Mediated Connection between Cell Wall Integrity and Competence in Streptococcus salivarius

**DOI:** 10.1128/msystems.00735-22

**Published:** 2022-11-07

**Authors:** Adrien Knoops, Alexandra Waegemans, Morgane Lamontagne, Baptiste Decat, Johann Mignolet, Jan-Willem Veening, Pascal Hols

**Affiliations:** a Louvain Institute of Biomolecular Science and Technology, Biochemistry and Genetics of Microorganisms, Université catholique de Louvain, Louvain-La-Neuve, Belgium; b Department of Fundamental Microbiology, Faculty of Biology and Medicine, University of Lausanne, Lausanne, Switzerland; University of Wisconsin-Madison

**Keywords:** cell-to-cell communication, genome-wide screen, quorum sensing, DNA transformation, ComRS, cell wall, CRISPRi, serine-threonine kinase

## Abstract

Competence is one of the most efficient bacterial evolutionary and adaptative strategies by synchronizing production of antibacterial compounds and integration of DNA released by dead cells. In most streptococci, this tactic is orchestrated by the ComRS system, a pheromone communication device providing a short time window of activation in which only part of the population is responsive. Understanding how this developmental process integrates multiple inputs to fine-tune the adequate response is a long-standing question. However, essential genes involved in the regulation of ComRS have been challenging to study. In this work, we built a conditional mutant library using CRISPR interference and performed three complementary screens to investigate competence genetic regulation in the human commensal Streptococcus salivarius. We show that initiation of competence increases upon cell wall impairment, suggesting a connection between cell envelope stress and competence activation. Notably, we report a key role for StkP, a serine-threonine kinase known to regulate cell wall homeostasis. We show that StkP controls competence by a mechanism that reacts to peptidoglycan fragments. Together, our data suggest a key cell wall sensing mechanism coupling competence to cell envelope integrity.

**IMPORTANCE** Survival of human commensal streptococci in the digestive tract requires efficient strategies which must be tightly and collectively controlled for responding to competitive pressure and drastic environmental changes. In this context, the autocrine signaling system ComRS controlling competence for natural transformation and predation in salivarius streptococci could be seen as a multi-input device integrating a variety of environmental stimuli. In this work, we revealed novel positive and negative competence modulators by using a genome-wide CRISPR interference strategy. Notably, we highlighted an unexpected connection between bacterial envelope integrity and competence activation that involves several cell wall sensors. Together, these results showcase how commensal streptococci can fine-tune the pheromone-based competence system by responding to multiple inputs affecting their physiological status in order to calibrate an appropriate collective behavior.

## INTRODUCTION

In the human digestive tract, bacteria face highly competitive pressure and physicochemical challenges. Surviving in this environment requires powerful and efficient strategies which must be tightly controlled and collectively coordinated (1–3). Quorum sensing (QS) devices are particularly suited to control concerted survival tactics since they perform bacterial density sensing. Although QS systems were initially thought to be restricted to this role, recent evidence suggests that QS systems can operate as autocrine modules and process multiple inputs (4). On the one hand, QS autocrine signaling allows heterogeneity amplification by positive feedback loops, a key feature for subpopulation activation (5–7). On the other hand, environmental stimuli can fine-tune the sensitivity of the pheromone-based apparatus (8, 9). This property is switching the QS system from a cell density to a multi-input device, integrating diverse stimuli to calibrate population-wide strategies (4).

One of the best-characterized QS-mediated process in Gram-positive bacteria is competence regulation (10). Orchestrating predation through bacteriocin production together with natural transformation, competence is regulated by two types of signaling systems in streptococci (11). The ComCDE system found in the mitis and anginosus groups relies on the sensing of the extracellular pheromone CSP (competence-stimulating peptide) that induces a phosphorelay leading to transcriptional activation of competence genes comprising *comX*, which codes for the master competence-specific sigma factor (12). The alternative predominant system in streptococci is based on the production/maturation of the pheromone XIP (*comX*-inducer peptide), which is internalized by the Opp transporter and binds the intracellular receptor ComR (13, 14). Subsequently, the dimeric ComR·XIP complex activates several bacteriocin and competence genes including *comX* (15–17).

Uncovering the environmental triggers allowing permissive conditions for competence QS has remained challenging in streptococci (18). Since two-component systems (TCS) and serine-threonine kinases (STK) are dedicated to sensing the outside world, they constitute attractive targets to couple environmental stimuli to QS reactivity. In Streptococcus pneumoniae, several of those sensors (e.g., StkP, CiaRH, VicRK) have been highlighted to control the ComCDE activity upon pH, O_2_, cell density, or antibiotic stresses (9, 19–23). In the cariogenic Streptococcus mutans species, other distal regulators have been highlighted, such as ScnRK, HdrM, BrsRM, CiaRH, or StkP, which link competence activation to various growth conditions (pH, carbohydrate source, oxygen, cell density) (24–31). In salivarius streptococci, we recently uncovered a regulatory inhibition by the CovRS environmental sensor of the ComRS signaling system (7). As exemplified by these three cases, despite the fact that environmental triggers can be shared, environmental sensors bridging detection of stimuli to competence can be highly divergent between species.

To investigate key sensors generating permissive conditions for competence activation, genome-wide screens are the fastest and best-suited approaches. While transposon insertion sequencing (Tn-seq) strategies have already revealed several regulators in S. mutans and S. pneumoniae (32, 33), classical knockout characterization of the identified genes is often impaired by their essentiality. Recently, a genome-wide CRISPR interference (CRISPRi) screening method was shown to overcome this drawback for Escherichia coli and S. pneumoniae (34–36). This technique combines the use of a guide RNA (gRNA) library targeting the whole genome with a catalytically dead mutant of Cas9 (dCas9), producing transcriptional interference upon DNA binding. Plugging in the dCas9 under the control of an inducible promoter allows the construction of a conditional mutant library which can be used for genetic screens and further for characterization of essential genes by knocking down their expression (34, 35).

In this work, we used this technique in combination with three distinct screens to unveil novel competence regulators. Cross-validation of the hits obtained from the three screens converged toward a connection between impairment of cell wall biogenesis and competence activation. Coherently, several sensors of the bacterial envelope integrity were identified, among which was StkP, suggesting a putative signaling pathway bridging cell wall stress to competence activation.

## RESULTS

### Screening for spontaneous transformation by genome-wide CRISPRi inhibition.

To identify unknown modulators of competence in Streptococcus salivarius HSISS4 (37), we set up a genome-wide CRISPRi strategy. To design gRNAs on the whole genome of HSISS4, we selected all the 20-nucleotide (nt)-long sequences followed by a protospacer adjacent motif (PAM; NGG sequence) on both DNA strands. For coding DNA sequences (CDSs), we retained only sequences displaying complementarity with the coding strand (nontemplate strand) (34). We ended up with a total of 83,103 gRNAs (see Data Set S1, sheet A, in the supplemental material) that were introduced under the control of a constitutive promoter (P_3_ [38]) at a neutral chromosomal locus. The random chromosomal distribution of gRNAs in the library was preliminarily evaluated by the direct sequencing of 40 individual clones (Fig. S1A).

10.1128/msystems.00735-22.1 FIG S1 Random chromosomal distribution of the gRNA library. (A) Forty randomly picked colonies from the gRNA library were PCR amplified, Sanger sequenced, and mapped on the Streptococcus salivarius HSISS4 genome. From outside to center, numbers denote the genomic position (× 10^5^ bp), and red and blue regions depict coding strand being on the (+) or (−) strand, respectively. Large regions empty of color correspond to clusters of tRNAs or rRNAs. Red and blue dots show the mapping of gRNAs targeting the (+) or (−) strands, respectively. Green dots show gRNAs targeting intergenic regions. (B) NGS mapping of the reads from gRNAs in the mock library (no library induction). The numbers of reads per gRNA are shown. gRNAs with common sequences were discarded since their mapping at multiple sites biases the analysis. Low-density mapping of gRNAs on the graph is associated with highly similar sequences such as rRNA or tRNA or multiple insertion of transposons. Removal of gRNAs with the same sequences from the analysis particularly influences the mapping in those regions. (C) Frequency distribution of gRNA counts from the mock library. Download FIG S1, TIF file, 2.3 MB. Copyright © 2022 Knoops et al. 2022 Knoops et al. https://creativecommons.org/licenses/by/4.0/ This content is distributed under the terms of the Creative Commons Attribution 4.0 International license.

10.1128/msystems.00735-22.8 DATA SET S1 List of oligonucleotides used for the CRISPRi strategy (sheet A), NGS normalized gene counts (sheets B to D), and gene-associated gRNA depletion scores (sheets E to G). Download Data Set S1, XLSX file, 10.1 MB. Copyright © 2022 Knoops et al. 2022 Knoops et al. https://creativecommons.org/licenses/by/4.0/ This content is distributed under the terms of the Creative Commons Attribution 4.0 International license.

**FIG 1:**
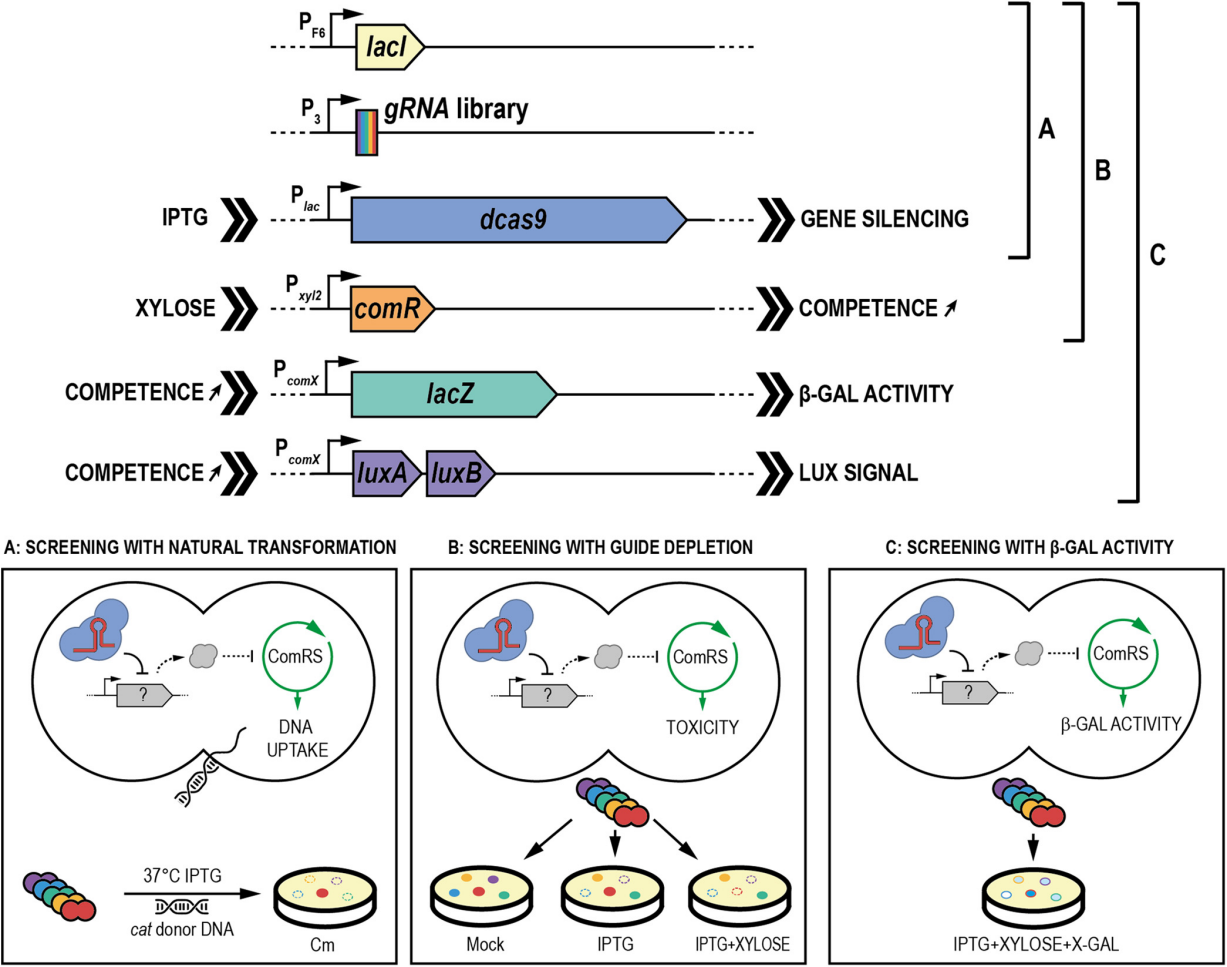
CRISPRi screening strategies for competence modulators in *S. salivarius.* A library of gRNAs was designed and introduced (P_3_*-gRNA*) in an engineered strain of *S. salivarius* harboring an IPTG-inducible system for dCas9 (P_F6_*-lacI*; P*_lac_-dcas9*). A first library was screened for spontaneous competence activation upon dCas9 inhibition by growing cells in chemically defined medium in the presence of IPTG and *cat* donor DNA. The selection on chloramphenicol plates was associated with inhibition of competence-negative players (A). A second library was generated by introducing the gRNA library into the same background with a supplemental construct consisting of a xylose-inducible promoter fused to *comR* (P*_xyl2_-comR*). The library was spread on control (mock), gRNA library-induced (IPTG), or gRNA library- and competence-induced (IPTG plus xylose) plates. NGS analysis of depleted gRNAs under the three conditions was performed to search for costly genes associated only with competence (B). A third library was built by adding *lacZ* under the control of P*_comX_* (P*_comX_-lacZ*) together with a competence luciferase reporter system (P*_comX_*-*luxAB*) to the previous strain and transferring the gRNA library into this background. The generated library was screened on plates containing IPTG, xylose, and X-Gal. gRNAs targeting potential competence-inhibitory or -activating genes were associated with dark blue or white phenotypes, respectively (C).

The transfer of the library was initially performed in a strain carrying an isopropyl-β-d-thiogalactopyranoside (IPTG)-inducible dCas9 (P_F6_*-lacI*; P*_lac_-dcas9* [35]), which was previously validated for generating CRISPRi conditional mutants (7) (Fig. 1). To evaluate the functionality of the library, this first strain was screened for the activation of spontaneous natural transformation. We hypothesized that dCas9-mediated repression of genes involved in competence inhibition (i.e., antagonist genes) will result in spontaneous natural transformation and donor DNA integration. We activated the interference library by adding IPTG (dCas9 activation) to a liquid culture supplemented with donor DNA containing a chloramphenicol resistance cassette (Fig. 1A). We were able to isolate 16 candidates after 3 independent rounds of selection, all harboring a different gRNA (Table 1). In order to confirm the phenotype generated by these gRNAs, we back-transformed them individually into the original strain and assessed their transformability. Spontaneous transformation was confirmed for 10 candidates (Table 1). Importantly, this functional screen succeeded in identifying two previously described negative effectors of competence acting on ComX or XIP stability (*clpC* and *pepF*, respectively) (39, 40).

**TABLE 1:** gRNA identification in spontaneous transformants

gRNA ID^*c*^	Genome position (bp)	Interference target	Gene name	Locus tag	Comment/function	Transformation rate
g_37	83910	Gene	*clpC*	HSISS4_00061	ComX degradation machinery ClpC	2.00E−06
g_38	85320	Gene	*clpC*	HSISS4_00061	ComX degradation machinery ClpC	6.00E−06
g_39	412742	Gene	*pepF*	HSISS4_00369	Oligoendopeptidase F	4.00E−06
g_27	1589000	Gene		HSISS4_01391	Bactoprenol glucosyltransferase	3.40E−02
g_30	1823312	Gene		HSISS4_01622	Hypothetical protein	2.00E−06
g_32	875880	Gene		HSISS4_00805	Hypothetical protein	5.80E−04
g_35	1442100	Gene		HSISS4_01302	Hypothetical protein	4.00E−06
g_33	Multiple sites	rRNA			16S rRNA	4.00E−06
g_34	Multiple sites	rRNA			16S rRNA	2.00E−06
g_36	Multiple sites	rRNA			16S rRNA	2.00E−06
g_26	112760	Gene	*gpmB*	HSISS4_00092	Phosphoglycerate mutase	ND^*a*^
g_42	499523	Gene	*carB*	HSISS4_00444	Carbamoyl synthase	ND
g_40^*b*^	1270227	Gene	*scuR*	HSISS4_01166	Intracellular receptor, bacteriocin-related communication system	ND
g_40^*b*^	1272924	Gene	*sarF*	HSISS4_01169	Intracellular receptor, bacteriocin-related communication system	ND
g_41	1775841	Gene	*pepXP*	HSISS4_01580	Dipeptidyl peptidase	ND
g_31	714120	Gene		HSISS4_00663	Extracellular nuclease 2	ND
g_43	664598	Intergenic				ND

a ND, not detected.

b g_40 gRNA recognizes both *scuR* and *sarF* genes.

c ID, identifier.

### Screening based on competence fitness cost.

**FIG 2:**
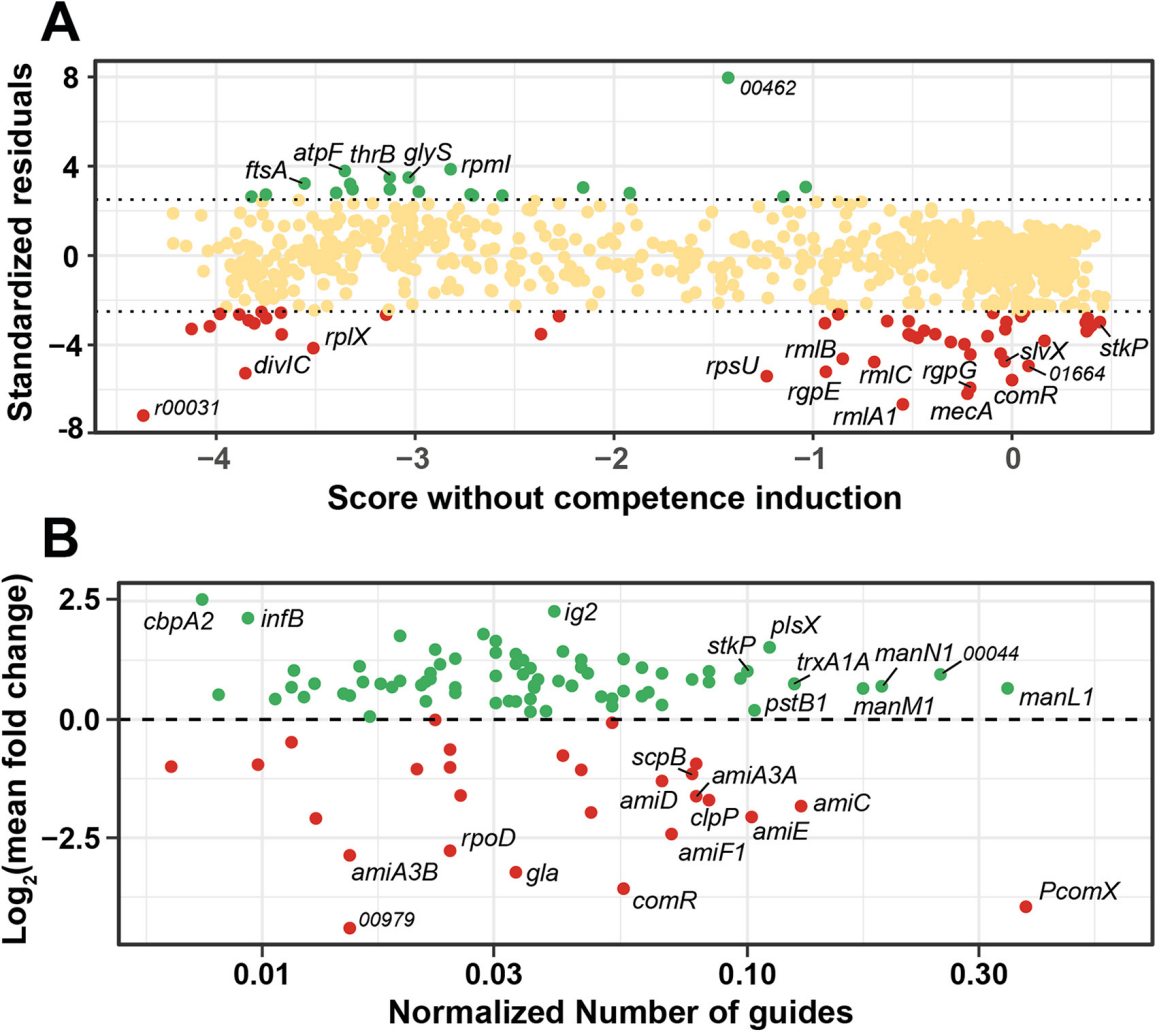
Selection of genes from CRISPRi screens. (A) gRNA depletion screen. The gRNA library was grown on M17G plates for ~12 generations with no induction (mock), with gRNA library induction (Ci), or with gRNA library and competence induction (Ci+C). The gRNAs (4 technical replicates per condition, ~40 million reads) were sequenced and mapped by using the MAGeCK algorithm. Using the same tool, we identified gRNA depletion in costly genes linked to library induction only (Ci versus mock) and both library and competence induction (Ci versus Ci+C) (Fig. S2). We then compared the gRNA depletion scores for each gene in both induction systems and performed a linear regression (Fig. S3). Standardized residuals of the regression were then computed and plotted in function of the score of each gene under the condition without competence induction (Ci). Positive (green) and negative (red) standardized residuals (arbitrary cutoff +2.5 and −2.5) denote genes with enriched or depleted gRNAs, respectively. Dots in yellow are considered nonsignificantly affected genes. (B) β-Gal screen. After library production (~10^5^ colonies), screening for dark blue and white clones on M17GL plates (with IPTG, xylose, and X-Gal; P*_comX_-lacZ*, P*_xyl2_*-*comR*), and validation with luciferase assays (P*_comX_-luxAB*), clones with the most dissimilar luciferase phenotypes (141 dark blue and 68 white clones) were sequenced for gRNA identification. The *y* axis displays the mean fold change log_2_ value of luciferase activity calculated on all the gRNAs targeting the same gene. The *x* axis displays the number of gRNAs targeting the same gene normalized by the expected total number of gRNAs present in the library for this gene. Green dots and red dots denote gene inhibition resulting in competence overactivation or repression, respectively.

The strategy of screening based on competence fitness cost was based on the burden of competence overactivation in the strain HSISS4 (16). We assumed that repression of competence-antagonist genes would produce a fitness cost, resulting in pool depletion of gRNAs targeting the corresponding genes. To set up this strategy, a second screening strain was generated by introducing a supplemental construct consisting of a xylose-inducible *comR* gene (P*_xyl2_*-*comR*), allowing a mild competence activation upon addition of xylose, a nonmetabolizable sugar in *S. salivarius* (7) (Fig. 1B). After introducing the gRNA library into this strain, we spread it under three different solid culture conditions. The first condition without any inducer was used as control (mock). The second condition was induced with IPTG alone to activate the CRISPRi library (Ci), and the third condition was supplemented with IPTG and xylose to concomitantly activate the CRISPRi library and competence (Ci+C). We hypothesized that we could identify modulator genes of competence by comparing the depletion of gRNAs between conditions Ci and Ci+C. To this aim, we performed high-throughput next-generation sequencing (NGS) to quantify each gRNA abundance per condition (Data Set S1, sheets B to D). We first evaluated the randomness and homogeneity of gRNA distribution without any selection pressure (mock) by visualizing the mapping of the gRNAs on the genome of HSISS4 (Fig. S1B). Validating our previous Sanger sequencing data (Fig. S1A), we showed that 99.7% (82,864 out of 83,104) of gRNAs were cloned in the noninduced library with an unbiased distribution (Data Set S1, sheet B; Fig. S1C). We next used the MAGeCK algorithm (41) to compare depletion of gRNAs between two conditions. As expected, the analysis of gRNA depletion between Ci and mock conditions uncovered well-known essential genes in streptococci (Data Set S1, sheet E; Fig. S2A), as well as competence-related genes (e.g., *covR*, *pepF*) whose inactivation was recently shown to be lethal in strain HSISS4 (7, 40). To avoid sampling bias, we also compared the Ci+C condition with the mock (Data Set S1, sheet F; Fig. S2B) and plotted against each other the scores obtained from the two comparisons with the mock (i.e., Ci versus mock and Ci+C versus mock) (Fig. S3). As expected, depletion scores in the two comparisons displayed a high correlation showing that gene fitness (i.e., positive, neutral, or negative) was conserved with or without competence activation (linear regression of *R*^2^ = 0.97). However, several outliers were present. Because they represent genes differentially affected between two conditions, we analyzed the standardized residuals (defined as the residuals of a regression model divided by their estimated standard deviation) of the linear regression (Fig. 2A; Data Set S2). We set up an arbitrary cutoff at +2.5 and −2.5 to identify the most representative outliers. Their statistical significance was confirmed by the direct comparison of the two conditions (Ci versus Ci+C; Data Set S1, sheet G). Several depleted gRNAs were found as targeting genes known as competence antagonists such as the *mecA* gene encoding the ComX adaptor of the Clp degradation machinery (standardized residuals < −2.5, Table 2) (42). Unexpectedly, many crucial genes for competence activation (*comR*, *amiACDEF*) or competence-based bacteriocin production/immunity (e.g., *slvX-HSISS4_01664* operon) also showed gRNA depletion (Table 2) (16). In the strain HSISS4, competence, bacteriocins, and bacteriocin-immunity genes are concomitantly activated through ComR (16). Therefore, those genes might have been selected because a reduced competence activation goes along with a lower immunity rate toward bacteriocins, ultimately leading to a high fitness cost. Indeed, since bacteriocin producers are present at high cell density on plates due to xylose-mediated competence activation, noncompetent and immunity-deficient cells will be killed through the well-established fratricide process (43).

**TABLE 2:** Identification of competence-costly genes from gRNA depletion screen

Gene category and name	Locus tag	Comment/function	Fitness cost score without competence induction^*a*^	Std residual (<−2.5)^*b*^
Competence related				
*comR*	HSISS4_00217	Competence intracellular receptor	0.00	−5.58
*amiF1*	HSISS4_01361	Oligopeptide ABC transporter, ATP-binding subunit F	−0.52	−2.94
*amiE*	HSISS4_01362	Oligopeptide ABC transporter, ATP-binding subunit E	−0.47	−3.68
*amiD*	HSISS4_01363	Oligopeptide ABC transporter, permease subunit D	−0.52	−3.53
*amiC*	HSISS4_01364	Oligopeptide ABC transporter, permease subunit C	−0.51	−3.59
*amiA3A*	HSISS4_01365	Oligopeptide ABC transporter, oligopeptide binding subunit A	−0.44	−3.37
	HSISS4_01664	SlvX immunity protein	0.08	−4.94
*slvX*	HSISS4_01665	Bacteriocin	−0.04	−4.73
*mecA*	HSISS4_00128	ComX degradation machinery adaptor protein	−0.22	−6.19
*spxA1*	HSISS4_00943	Transcriptional regulator	0.38	−2.80
Cell envelope related				
*stkP*	HSISS4_01348	Serine-threonine kinase	0.44	−2.98
*acpP1*	HSISS4_00021	Acyl carrier protein	0.06	−2.54
*rgpG*	HSISS4_00129	Polysaccharide synthesis protein	−0.21	−5.92
*rgpF*	HSISS4_01378	Polysaccharide synthesis protein	−0.21	−4.43
*rgpE*	HSISS4_01379	Extracellular rhamnan synthesis protein	−0.94	−5.20
*rgpA2*	HSISS4_01383	Extracellular rhamnan synthesis protein	−2.37	−3.52
*rmlA1*	HSISS4_00723	Rhamnose synthesis protein	−0.55	−6.66
*rmlC*	HSISS4_00724	Rhamnose synthesis protein	−0.69	−4.77
*rmlB*	HSISS4_00725	Rhamnose synthesis protein	−0.85	−4.62
*pgmA*	HSISS4_01102	Phosphoglucomutase	−0.12	−3.61
*dgk*	HSISS4_00536	Lipid carrier recycler	−2.28	−2.71
*murG*	HSISS4_00684	Peptidoglycan lipid II precursor synthesis	−3.98	−2.62
	HSISS4_00889	Exporter of O-antigen, teichoic acids, lipoteichoic acids (WpsG)	−0.24	−3.97
*dltD*	HSISS4_01108	Poly(glycerolphosphate chain) d-alanine transfer protein	−0.03	−2.98
*dltC*	HSISS4_01109	d-Alanine phosphoribitol ligase subunit 2	−0.48	−3.68
*dltB*	HSISS4_01110	d-Alanyl transfer protein	−0.31	−3.87
*dltA*	HSISS4_01111	d-Alanine phosphoribitol ligase subunit 1	−0.39	−3.52
*dltX*	HSISS4_01112	d-Ala-teichoic acid biosynthesis protein	−0.63	−2.93
*pstB1*	HSISS4_00936	Phosphate transport, ATP-binding protein	0.37	−3.00
*pstC2*	HSISS4_00937	Phosphate transport, permease protein	0.38	−3.39
*pstC1*	HSISS4_00938	Phosphate transport, permease protein	0.38	−3.27
*pstS*	HSISS4_00939	Phosphate transport, phosphate binding protein	0.40	−3.14
*divIC*	HSISS4_00008	Cell division protein	−3.85	−5.27
*ftsL*	HSISS4_01598	Cell division protein	−4.12	−3.29
Translation				
*prfB*	HSISS4_00848	Peptide chain release factor	−0.06	−4.39
*proS*	HSISS4_00152	Prolyl tRNA synthetase	−3.77	−2.53
*rplM*	HSISS4_00076	Large subunit ribosomal protein	−3.67	−3.53
*rplX*	HSISS4_01812	Large subunit ribosomal protein	−3.51	−4.15
*rpsU*	HSISS4_01396	Small subunit ribosomal protein	−1.23	−5.40
*rpsF*	HSISS4_01661	Small subunit ribosomal protein	−3.81	−3.04
*rpsE*	HSISS4_01806	Small subunit ribosomal protein	−3.88	−2.64
*rpsS*	HSISS4_01819	Small subunit ribosomal protein	−3.84	−2.90
	HSISS4_00271	Ribosomal protein	−3.75	−2.80
	HSISS4_r00031	tRNA Met	−4.37	−7.16
	HSISS4_r00059	tRNA Glu	−4.03	−3.17
	HSISS4_r00070	tRNA Arg	−3.67	−2.56
Other				
*ctsR*	HSISS4_00060	Stress transcriptional regulator	−0.09	−2.57
*atpE*	HSISS4_00399	ATP synthase	−3.15	−2.65
*pyrH*	HSISS4_00354	Uridine monophosphate kinase	−0.94	−3.03
*sipA*	HSISS4_01673	Secretory signal peptidase	−0.87	−2.63
	HSISS4_00898	Permease	0.05	−2.73
	HSISS4_00523	Hypothetical protein	0.16	−3.82
	HSISS4_00888	Hypothetical protein	−0.03	−3.30

a Fitness-cost scores were computed with the MAGeCK algorithm by comparing the total depletion of gRNAs per gene under the mock condition with that under the library-induced condition (Ci).

b Standardized (Std) residuals (cutoff value of <−2.5) were calculated as the deviation from the linear regression performed with the fitness-cost scores for conditions with library induction (Ci) and with library induction together with competence induction (Ci+C).

10.1128/msystems.00735-22.2 FIG S2 Gene-associated gRNA depletion scores. gRNA reads computed with library induction (A) and with library induction plus competence activation (B) were both compared to the reads computed under the mock condition by using the MAGeCK algorithm. The algorithm generated a score translating the total depletion of gRNAs for one gene and a false-discovery rate (FDR) value, as a significant marker of the score. The plots show the score computed for each gene (red, FDR > 0.05; blue, FDR < 0.05). Each gene was associated with a random number (gene index) for the sake of clarity. Values for each gene can be found in Data Set S1, sheets E and F. Download FIG S2, TIF file, 2.0 MB. Copyright © 2022 Knoops et al. 2022 Knoops et al. https://creativecommons.org/licenses/by/4.0/ This content is distributed under the terms of the Creative Commons Attribution 4.0 International license.

10.1128/msystems.00735-22.3 FIG S3 Linear regression of gene-associated gRNA depletion scores. gRNA depletion values for each gene in comparison to the mock condition (Fig. S2) are plotted against each other. The linear regression was computed by using the *lm* function from R using QR decomposition (*R*^2^ = 0.97). Dashed lines denote a score of zero associated with a neutral fitness effect of gene inhibition. Download FIG S3, TIF file, 0.9 MB. Copyright © 2022 Knoops et al. 2022 Knoops et al. https://creativecommons.org/licenses/by/4.0/ This content is distributed under the terms of the Creative Commons Attribution 4.0 International license.

10.1128/msystems.00735-22.9 DATA SET S2 List of competence-associated genes (standardized residuals) from the gRNA depletion screen. Download Data Set S2, XLSX file, 0.1 MB. Copyright © 2022 Knoops et al. 2022 Knoops et al. https://creativecommons.org/licenses/by/4.0/ This content is distributed under the terms of the Creative Commons Attribution 4.0 International license.

### Screening based on transcriptional activity of the *comX* promoter.

The last screening strategy was based on β-galactosidase (β-Gal) activity that allows the colorimetric evaluation of competence activation level in individual clones on plates with low selective pressure. For this purpose, a strain was generated by plugging the promoter of *comX* in front of the native *lacZ* gene (P*_comX_-lacZ*) together with a P*_comX_* luciferase (Lux) reporter system (P*_comX_-luxAB*) into the dCas9 and xylose-inducible competence strain (Fig. 1C). We transformed the gRNA library into this genetic background and spread it onto M17GL plates supplemented with IPTG, xylose, and X-Gal (5-bromo-4-chloro-3-indolyl-β-d-galactopyranoside) for detection of β-Gal activity. We examined ~94,000 isolated colonies, searching for white and dark blue phenotypes. While white phenotype is associated with targeted genes required for competence activation, dark blue phenotype is related to targeted genes repressing competence development. We next reisolated the selected colonies to confirm their phenotypes and ended up with 141 dark blue and 68 white clones. We sequenced their gRNAs to identify the interference target and quantified their inhibition effects on P*_comX_* activation by using the P*_comX_*-*luxAB* module present in the strain. To this aim, we slightly overexpressed *comR* with xylose by using the P*_xyl2_-comR* module and induced the gRNA-based inhibition system by adding IPTG. We compared the specific luciferase activity of all the selected clones to that of the initial strain harboring no gRNA. The sequences of the gRNAs, their identified targets, and their fold changes in P*_comX_* activation are displayed in Data Set S3, sheet A.

10.1128/msystems.00735-22.10 DATA SET S3 List of gRNA/targeted genes (sheet A) and normalized competence-associated genes (sheet B) from the β-Gal screen. Download Data Set S3, XLSX file, 0.03 MB. Copyright © 2022 Knoops et al. 2022 Knoops et al. https://creativecommons.org/licenses/by/4.0/ This content is distributed under the terms of the Creative Commons Attribution 4.0 International license.

Since both frequency of selected gRNAs targeting the same gene and fold change in P*_comX_* activation were relevant features to identify new competence regulators, we combined those two parameters in the same analysis. On one hand, we calculated the mean fold change in P*_comX_* activation for all gRNAs targeting the same gene. On the other hand, we counted the number of selected gRNAs targeting the same gene. In addition, we normalized the count by the total expected number of gRNAs of the library targeting each defined gene to avoid any gene-size bias (higher probability to encounter a gRNA targeting a larger gene) (Data Set S3, sheet B). We plotted those two variables (activation fold change versus normalized gRNA frequency) and validated the screen by finding most of the proximal effectors of the ComRS system, i.e., *comR*, *amiACDEF*, *clpC*, and P*_comX_* (Fig. 2B and Data Set S3, sheet B) (7, 16, 42). We next applied an arbitrary cutoff [normalized count > 0.02 and log_2_(FC) > 0.5] to find the most significant genes with an antagonist function toward competence (Table 3). We thereby selected several genes whose role in competence inhibition was also suggested with the gRNA depletion screen, such as the phosphate transporter system (*pstC1*) and the serine-threonine kinase (*stkP*) genes. Strikingly, the mannose/glucosamine phosphotransferase (PTS) transporter operon (*manLMN*) was particularly overrepresented, even though absent from the two previous screens. This result might be an artifactual consequence of a carbon metabolism shift enhancing xylose uptake ultimately resulting in higher *comR* induction but could also be due to a link between mannose catabolism and competence as reported in S. mutans (30).

**TABLE 3:** Identification of competence-associated antagonist genes from β-Gal screen

Gene category and name	Locus tag	Comment/function	Normalized count (>0.01)^*a*^	Mean log_2_(FC) Lux (>0.5)^*b*^
Competence related^*c*^				
*hk13*	HSISS4_01230	Histidine kinase	0.03	1.80
*manL1*	HSISS4_00257	PTS system, mannose-specific IIAB component	0.34	0.66
*manM1*	HSISS4_00256	PTS system, mannose-specific IIC component	0.17	0.66
*manN1*	HSISS4_00255	PTS system, mannose-specific IID component	0.19	0.70
*med*	HSISS4_01089	Nucleoside-binding protein	0.06	0.57
Cell envelope related				
*stkP*	HSISS4_01348	Serine-threonine protein kinase	0.10	1.02
*LiaF*	HSISS4_01346	LiaSR-associated transporter	0.04	1.08
*plsX*	HSISS4_00020	Phosphate:acyl-acyl carrier protein (ACP) acyltransferase	0.11	1.52
	HSISS4_01826	Acyltransferase family	0.04	0.72
*murJ*	HSISS4_00717	Lipid II flippase	0.06	1.28
*murC*	HSISS4_00190	UDP-*N*-acetylmuramate-alanine ligase	0.04	0.81
*murZ*	HSISS4_01465	UDP-*N*-acetylglucosamine 1-carboxyvinyltransferase	0.03	1.41
*glmS*	HSISS4_01060	Glucosamine–fructose-6-phosphate aminotransferase isomerizing	0.02	0.80
*gcaD*	HSISS4_00481	*N*-Acetylglucosamine-1-phosphate uridyltransferase/glucosamine-1-phosphate *N*-acetyltransferase (GlmU)	0.02	0.86
*rgpX3*	HSISS4_01386	Heteropolysaccharide repeat unit export protein	0.04	1.44
	HSISS4_00330	Lipopolysaccharide biosynthesis protein	0.04	0.85
*dltA*	HSISS4_01111	d-Alanine–poly(phosphoribitol) ligase subunit 1	0.04	0.67
*dltB*	HSISS4_01110	d-Alanyl transfer protein	0.02	1.17
*pstC1*	HSISS4_00938	Phosphate transport system permease protein	0.03	1.29
*pgmA*	HSISS4_01102	Phosphoglucomutase	0.05	0.98
*asp3*	HSISS4_01316	Accessory secretory protein	0.04	0.71
*pcsB2*	HSISS4_00358	GBS^*d*^ surface immunogenic protein	0.02	1.48
Amino acid metabolism				
*sdaA*	HSISS4_01162	l-Serine dehydratase, alpha subunit	0.05	1.26
*argJ*	HSISS4_00385	Glutamate *N*-acetyltransferase/*N*-acetylglutamate synthase	0.02	0.98
*pepP*	HSISS4_01648	Aminopeptidase P	0.06	1.10
*pepS*	HSISS4_00051	Aminopeptidase S	0.02	0.73
*gnlP*	HSISS4_01405	Glutamine ABC transporter/glutamine-binding permease	0.04	0.77
*livG2*	HSISS4_00477	ABC-type multidrug transport system, ATPase component	0.06	0.60
*livJ*	HSISS4_00287	High-affinity leucine-specific transport system	0.03	1.38
	HSISS4_00832	Glutamate transport membrane-spanning protein	0.03	1.26
	HSISS4_00833	Glutamate transport permease protein	0.05	1.10
Other				
*galR*	HSISS4_01243	Galactose operon repressor	0.03	0.68
	HSISS4_01867	Transcriptional regulator, PadR family	0.08	1.02
*nusA*	HSISS4_00269	Transcription termination protein	0.03	1.66
*cshA*	HSISS4_01831	Chromosome segregation helicase	0.03	0.56
	HSISS4_00847	Epoxyqueuosine (oQ) reductase	0.02	0.69
*gidA*	HSISS4_01879	tRNA uridine 5-carboxymethylaminomethyl modification enzyme	0.02	0.79
	HSISS4_01426	Acetyltransferase	0.07	0.97
	HSISS4_01531	RNA-binding protein	0.08	0.79
*trxA1A*	HSISS4_00080	Thioredoxin	0.13	0.76
*dnaK1*	HSISS4_00097	Chaperone protein	0.02	1.77
*scrK*	HSISS4_01640	Fructokinase	0.10	0.87
*tpiA*	HSISS4_00409	Triosephosphate isomerase	0.08	0.85
*purF*	HSISS4_00024	Amidophosphoribosyltransferase	0.02	0.76
*pyrDb*	HSISS4_00974	Dihydroorotate dehydrogenase, catalytic subunit	0.02	1.13
	HSISS4_01010	Phenazine biosynthesis-like protein	0.13	0.75
	HSISS4_00044	Hypothetical protein	0.25	0.95
	HSISS4_00614	Hypothetical protein	0.03	0.92
	HSISS4_01307	Hypothetical protein	0.03	0.95
	IG2	Large intergenic region (position 130920)	0.04	1.25
	IG3	Large intergenic region (position 46047)	0.067	1.36
	IG6	Large intergenic region (position 816162)	0.033	0.84

a Normalized counts (cutoff value of >0.01) were calculated by dividing the number of gRNAs targeting one gene by the expected number of gRNAs targeting this gene in the library.

b Mean of the log_2_ fold change is an average of all the fold changes in specific Lux activity for the different gRNAs targeting the same gene (cutoff value of >0.5).

c *hk13*, *manLMN*, and *med* genes were previously reported as involved in competence regulation in *S. salivarius*, S. mutans, and B. subtilis, respectively (7, 30, 76).

d GBS, group B Streptococcus.

### Cell wall integrity is a signal for competence.

**FIG 3:**
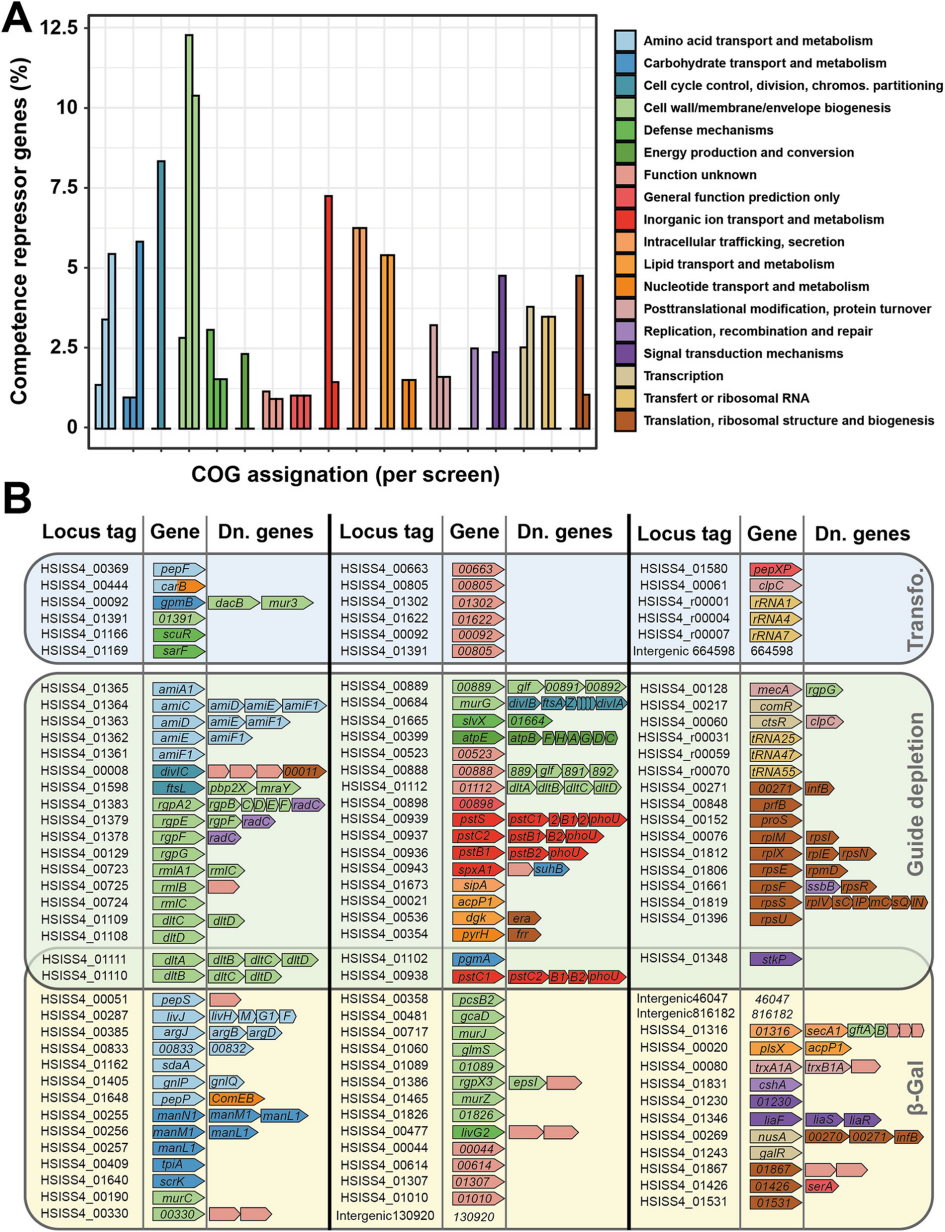
Functional assignation of competence repressor genes from CRISPRi screens. (A) Relative abundance of COG-assigned genes. A COG assignment was associated with every gene from the HSISS4 genome. For each COG type, the proportion (percentage) of selected genes with a defined screen was calculated against all the genes with this COG assignment of the genome. This proportion is displayed per screen (first bar, transformation screen; second bar, gRNA depletion screen; third bar, β-Gal screen). (B) Details of all selected genes displayed per screen. Operons (Dn. genes) are shown since CRISPRi also silences downstream genes. Genes are colored according to their COG assignment.

Since many genes were identified to affect competence by the three screening approaches, we used the Clusters of Orthologous Genes (COG) database (44) to classify them by general function. For this analysis, we selected only the genes whose inhibition is expected to induce competence [i.e., all the genes from the transformation screen, genes with standardized residuals of <−2.5 from the growth screen, and genes with log_2_(FC) of >0.5 and normalized count of >0.02 from the colorimetric screen]. We next assessed the importance of the different pathways for competence activation. For this purpose, we counted the number of genes per screen involved in one COG function and normalized it by the total number of genes within this COG function in the HSISS4 genome (Fig. 3A). This analysis indicated that the most-represented function was cell wall/membrane/envelope biogenesis (23% of all the genes highlighted versus ~5% at the whole-genome level). Furthermore, we observed that overlapping genes between gRNA depletion and β-Gal screens were all directly or indirectly involved in cell envelope assembly. Indeed, we identified in both screens the *dltA* and *dltB* genes involved in teichoic acid d-alanylation (45), the phosphoglucomutase *pgmA* gene involved in the biosynthesis of extracellular polysaccharides (46), the cell wall-related serine-threonine kinase *stkP* gene (47, 48), and the phosphate transporter *pstC1* gene with an important role for poly(glycerophosphate) teichoic acid synthesis (49) (Fig. 3B).

**FIG 4:**
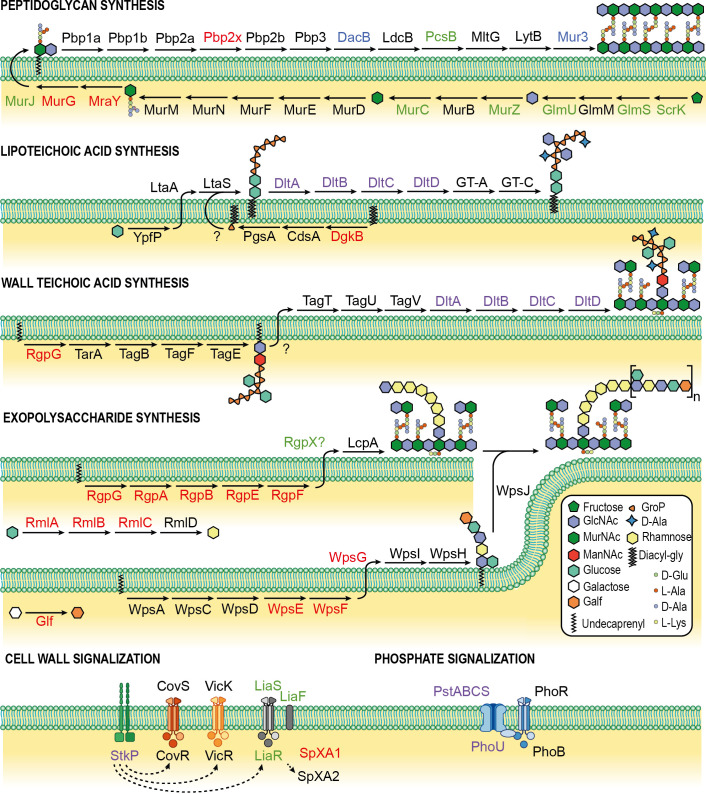
Cell wall pathways and competence negative modulators from CRISPRi screens. Major cell wall biosynthesis and signalization pathways are depicted. Proteins selected by the transformation, gRNA depletion, and β-Gal screens are shown in blue, red, and green, respectively. Proteins selected in both gRNA depletion and β-Gal screens are shown in light violet. In the absence of literature for complete reconstructed pathways, lipoteichoic acid synthesis is based on knowledge from Staphylococcus aureus, wall teichoic acid synthesis is based on knowledge from B. subtilis 168, and polysaccharide synthesis is based on knowledge from Lactococcus lactis. HSISS4_00889, 00890, 00891, and 00892 were renamed with Lactococcus lactis homologs WpsG, Glf, WpsE, and WpsF, respectively. GlcNAc, *N*-acetylglucosamine; MurNAc, *N*-acetyl muramic acid; ManNAc, *N*-acetylmannosamine; Galf, galactofuranose; Diacyl-gly, diacyl-glycerol; GroP, glycerol-phosphate.

We next drew a more precise view of the different cell wall pathways targeted by gRNA that presumably lead to competence activation. We found that genes involved in the synthesis of peptidoglycan, teichoic acids, and extracellular polysaccharides were all affected (Fig. 4). In parallel, key sensors (StkP, LiaFSR) or mediators (SpxA1) known to be triggered by cell wall alterations were also identified in the screens (24, 50–54), suggesting a possible link between cell wall integrity and ComRS activation.

### StkP controls *comX* expression through muropeptide binding.

Since StkP was highlighted in two screens with multiple different gRNAs and is cell wall associated, we decided to further investigate its link with competence activation. Serine-threonine kinases are pleiotropic regulators that control key cellular processes such as dormancy, virulence, cell division, and cell wall synthesis through protein phosphorylation (47, 48). In *S. salivarius*, only one serine-threonine kinase homolog is present and displays PASTA motifs shown to bind muropeptides in Bacillus subtilis (55). Besides, StkP of S. pneumoniae has been shown to coordinate cell wall synthesis and septation (24, 56–58) while an unclear link with competence has been suggested in S. mutans and S. pneumoniae (19, 23, 24).

**FIG 5:**
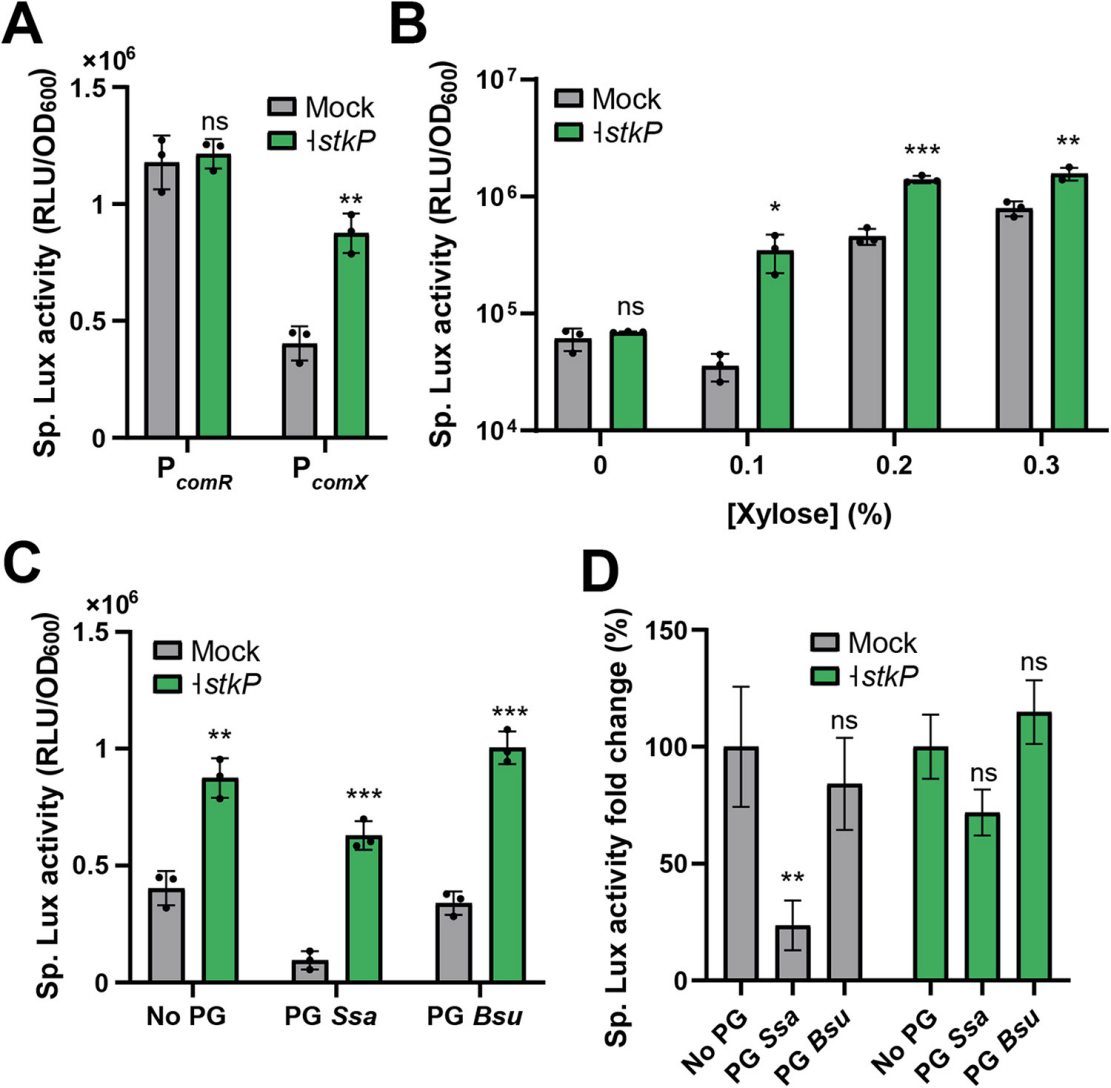
StkP controls *comX* expression by sensing peptidoglycan extracts. (A) Effect of *stkP* inhibition on *comR* and *comX* expression. A dCas9 module (P_F6_*-lacI*; P*_lac_-dcas9*) together with a gRNA targeting *stkP* (P_3_-*g_23*) was used to inhibit *stkP* transcription. The dCas9 interference system was associated with a P*_comR_*-*luxAB* or a P*_comX_-luxAB* reporter fusion together with a xylose-inducible *comR* module (P*_xyl2_-comR*). Mock denotes the same strain without dCas9 interference. (B) Effect of ComR level on StkP-mediated activation of *comX*. The P*_comX_-luxAB* P*_xyl2_-comR* strain (described for panel A) was incubated with various xylose concentrations (0, 0.1, 0.2, 0.3%) with or without *stkP* inhibition. (C) Effect of peptidoglycan (PG) extracts on StkP-mediated activation of *comX*. PG extracts of *S. salivarius* (PG *Ssa*) or B. subtilis (PG *Bsu*) were added to a culture of the P*_comX_-luxAB* strain (described in panel A) at a final concentration of 0.3 mg/mL. (D) Specific Lux activity (%) calculated between the culture with no addition of PG extracts (No PG, 100%) and the related condition. Percentages were calculated with the data presented in panel C. For P*_comX_-luxAB* activation, 0.25% xylose was used unless stated otherwise. For CRISPRi *stkP* inhibition, 1 mM IPTG was used. Dots denote technical triplicate values for mock and biological triplicate values for mutants, ± standard deviation. A statistical *t* test was performed for each condition in comparison to related control (Ctrl, mock; panels A, B, and D) or one-way ANOVA with Dunnett’s test for multiple comparison (Ctrl, no PG; panel E). ns, nonsignificant, *P* > 0.05; *, *P* < 0.05; **, *P* < 0.01; ***, *P* < 0.001.

In a first set of experiments, we transferred a gRNA targeting *stkP* in a strain harboring the dCas9 module (P_F6_-*lacI* P*_lac_-dcas9*) together with a luciferase reporter of the transcriptional activity of *comR* (P*_comR_-luxAB*) or *comX* (P*_comX_-luxAB*) and the xylose-inducible module allowing competence activation (P*_xyl2_-comR*). Monitoring activation of those two promoters with or without *stkP* inhibition suggested that StkP level influences *comX* expression but has no impact on *comR* transcription (Fig. 5A). We next used the same *comX* reporter strain with increasing xylose concentrations for *comR* induction and measured P*_comX_* activation with or without *stkP* inhibition (Fig. 5B). Stronger inhibitions of *stkP* were recorded for lower ComR levels, suggesting that ComR overproduction interferes with the StkP-mediated regulation of *comX*.

Since StkP was shown to bind specific muropeptides via its PASTA domains (55), we investigated if the addition of peptidoglycan extracts was able to modulate competence. To this aim, we purified peptidoglycan from *S. salivarius* (l-Lys pentapeptide) or B. subtilis (*meso*-diaminopimelic acid [DAP] pentapeptide) and measured the activation of the P*_comX_-luxAB* reporter strain with (-|*stkP*) or without (mock) dCas9 interference on *stkP* expression (Fig. 5C). While *S. salivarius* peptidoglycan could decrease P*_comX_* activation, B. subtilis extracts (negative control) did not result in a significant reduction. Moreover, adding peptidoglycan from *S. salivarius* prevented P*_comX_* repression when *stkP* was inhibited, suggesting that StkP mediates the signalization (Fig. 5D).

Altogether, these results suggest that StkP interferes with the transcriptional activity of the ComR·XIP complex by an unknown mechanism, which is modulated by the binding of specific muropeptides.

## DISCUSSION

**FIG 6:**
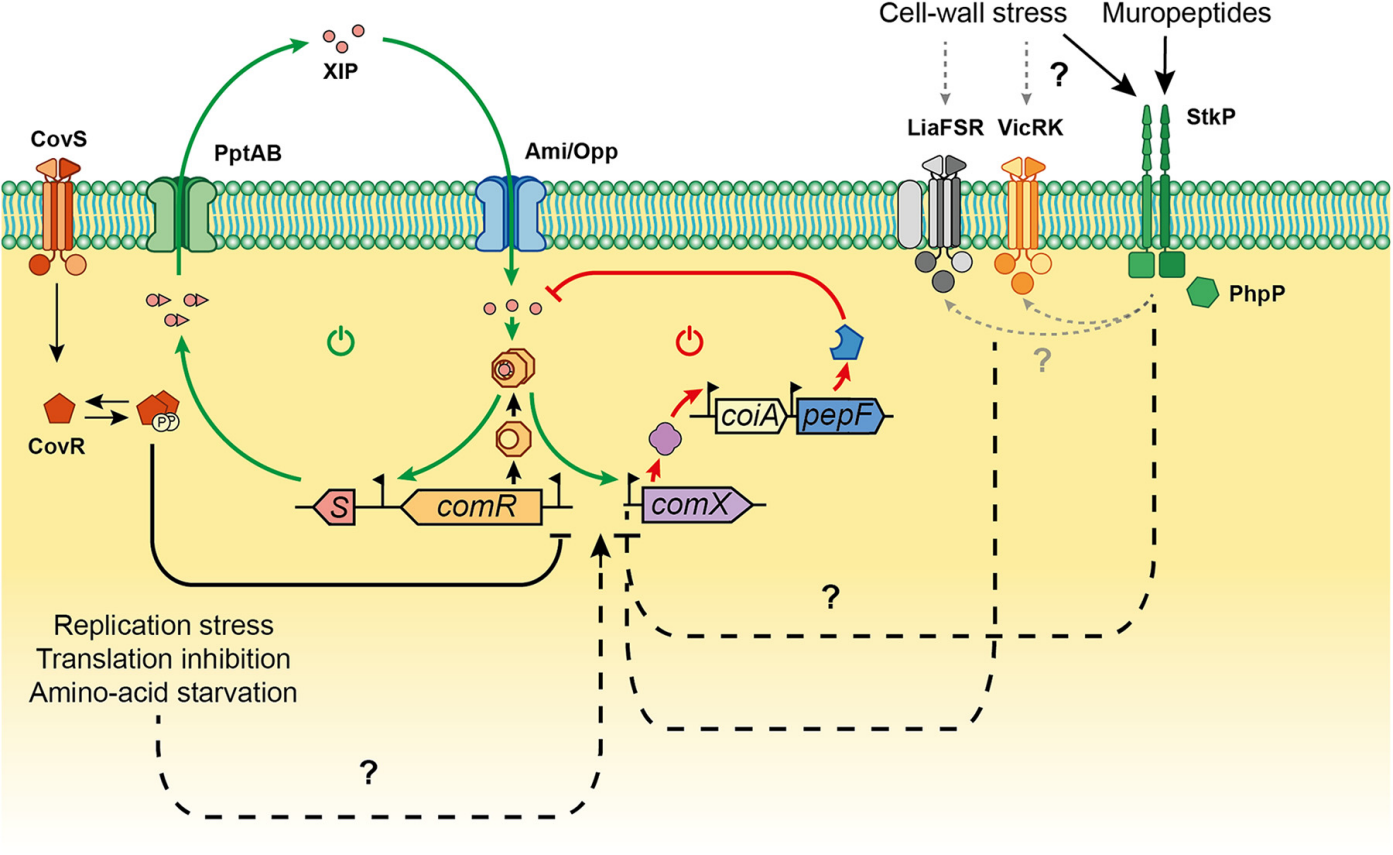
Model of competence regulation integrating cell wall sensors and physiological stresses in *S. salivarius*. Upon CovRS repression release, ComR reaches a threshold concentration allowing the activation of a positive feedback loop (green arrows, power-on icon). The positive loop is triggered by XIP binding to ComR, producing the ComR·XIP complex which activates *comS* transcription. ComS is then exported by the transporter PptAB and matured. The mature XIP pheromone can then enter the cell by the oligopeptide generic transporter Ami/Opp and bind ComR to enhance the loop. In parallel, the ComR·XIP complex will trigger the transcription of *comX*, encoding the central regulator of competence. This will activate all the late genes necessary for natural transformation including the *coiA-pepF* operon. PepF accumulation will result in XIP degradation, generating a negative feedback loop (red arrows, power-off icon) on the ComRS system, ultimately leading to competence exit. In parallel, cell wall stress and/or free muropeptides can be sensed by the serine-threonine kinase StkP, LiaFSR, and VicRK to modulate the transcriptional activation of *comX*, most probably via interfering with the activity of the ComR·XIP complex. Other physiological stresses such as replication stress, translation inhibition, or amino acid starvation were also identified as conditions that could activate competence development.

How QS modules integrate multiple inputs to fine-tune their sensitivity and optimize collective behavior is a challenging topic. In this work, we performed a genome-wide screen coupled to three different readouts to uncover key triggers of ComRS-mediated competence activation. Using a conditional mutant library, we highlighted a connection between cell wall biogenesis and competence activation. Moreover, we uncovered a link between muropeptide sensing via the serine threonine StkP and competence development. Those pieces of evidence suggest a key role of cell wall stress in the competence response (Fig. 6).

To discover novel players involved in competence regulation, we built a CRISPRi-based library and performed three types of screening in parallel. The interference technology offers several advantages over the classical random transposon mutagenesis (59), but the primary one is the production of conditional mutants allowing the study of essential/deleterious genes. Considering the transformation screen, the library was transiently induced, dampening the toxicity-acquired phenotype due to constitutive activation of natural transformation. This strategy provided a direct screening method for DNA integration and allowed us to select gRNAs targeting essential genes among which was *pepF*, a gene essential for competence shutoff recently discovered in *S. salivarius* (40). In addition, we also selected two different gRNAs targeting *clpC*, a gene encoding a component of the MecA-ClpCP machinery responsible for ComX degradation (39, 60). Those results confirm the roles of PepF and ClpC to prevent spontaneous competence activation at the early and late stage of competence, respectively (40, 42, 60). Moreover, novel competence modulators were identified such as a putative bactoprenol glucosyltransferase and 3 hypothetical proteins. Specifically, interference on the putative bactoprenol glucosyltransferase resulted in a high transformation rate (~10^−2^, Table 1), suggesting an important role of this player for competence control. Although the transformation screen displays interesting features to select essential genes connected to competence development, it would require a massive number of cells to ensure a complete coverage of the high-density gRNA library. This issue is not present in the gRNA depletion screen, where high-throughput NGS is exploited to map and quantify all the gRNAs, generating a complete picture at the genome scale. Nevertheless, the identification of the genes is based on the competence-related toxic phenotype. This feature could limit the detection of essential genes whose inhibition has a high fitness cost. Of note, the competence-associated toxicity used in the gRNA depletion screen could explain some intriguing results. While NGS data showed a depletion of gRNAs targeting genes involved in the downregulation of competence such as *mecA*, the depletion of gRNAs targeting crucial genes for competence activation (e.g., *comR* or the *ami/opp* operon) was counterintuitive. To reconcile these findings, we reasoned that a lack of functional competence goes along with an impairment in bacteriocin immunity. Consequently, the gRNA depletion will also include bacteriocin/immunity loci and key players required for competence activation (Table 2 and see Data Set S2 in the supplemental material). Finally, as the colorimetric β-Gal test is based on P*_comX_* activity and visual selection, this screen drastically reduces any fitness bias. To sum up, this work highlights the added value of combining different screening approaches to unveil the largest set of candidate genes connected to competence control.

The three screens converge to select gRNAs involved in key envelope biogenesis processes and its control by cell wall sensors (Fig. 4). The connection between cell wall and competence has been reported only in a similar experiment with Tn-seq in S. mutans (61). However, the authors report that inactivation by transposon insertion of the cell wall-related genes *pknB* (homolog of *stkP*), *rgpL*, *dltA*, and *liaS* results in a lower activation of competence, contrasting with the results obtained in this study. Opposite effects of competence regulators in *S. salivarius* and S. mutans have already been reported for the CovRS system (7, 62) and showcase that species have evolved control mechanisms in line with their own lifestyles. Aside from the cell wall synthesis, several other pathways were highlighted (Fig. 6). One of them is translation, with several important players targeted (rRNAs, tRNAs, peptide chain release factor, ribosomal proteins, and tRNA synthetases). This correlation is interesting in the light of the work of Stevens et al., who showed that translation fidelity impairment promotes competence activation in S. pneumoniae (63). In addition, important genes involved in chromosome replication/segregation (*priA*, *cshA/rarA*, *scpB*) and DNA repair (*mutL*, *mutT*, *dinP*) were also underlined by the screens (Table 3; Data Sets S2 and S3, sheet B). Replication stress was previously shown to induce pneumococcal competence, but the exact mechanism remains unclear and involves *comCDE* gene dosage control and/or a role for arrested and unrepaired replication forks (64, 65). The screens did highlight a role for enzymes or transporters involved in amino acid biosynthesis or uptake for arginine (CarB and ArgJ), glutamine (GlnP), glutamate (HSISS4_00833 and 00832), and leucine (LivJ). Amino acid starvation is known to trigger the stringent response via RelA and the production of (p)ppGpp alarmones (66), which was shown to influence competence regulation in S. mutans (67). Altogether, the screens performed here suggest that *S. salivarius* competence control relies on the sensing of various alterations of key metabolic/physiological functions, reinforcing the view that competence activation could be seen as a general stress response in streptococci.

In this work, we specifically investigate StkP, a key sensor of cell wall integrity in S. pneumoniae (54). In streptococci, StkP was also shown to phosphorylate classical response regulators of two-component systems such as the virulence regulator CovR in Streptococcus agalactiae and Streptococcus pyogenes (68, 69), the cell wall regulator VicR in S. mutans and S. pneumoniae (24, 54), and the competence regulator ComE in S. pneumoniae, for which StkP phosphorylation triggers a distinct regulon from the aspartate phosphotransfer mediated by ComD (22). The pleiotropic effects of StkP and its involvement in major cellular processes are probably the reason why its impact on competence has been reported but remains controversial in S. pneumoniae (19, 22, 23). In *S. salivarius*, we showed that *stkP* depletion promotes a higher *comX* activation without major effect on *comR* expression. This suggests a mechanism acting directly on the ComR sensor by increasing its transactivator properties. This hypothesis is strengthened by the fact that ComR overexpression curtails the effect of StkP on *comX* activation (Fig. 5B). The exact process remains to be discovered, even if it suggests a direct effect of StkP on ComR. Two nonexclusive mechanisms could explain the control of competence by StkP in *S. salivarius*. On one hand, the kinase could sense directly or indirectly a disfunction in the cell wall synthesis. Besides a direct effect on ComR, this impairment could also be transmitted to other cell wall sensors. Interestingly, two of these sensor systems (i.e., VicRK and LiaSRF) were highlighted in the β-Gal screen (Table 3 and Data Set S3, sheet B) and were previously shown to affect competence activation in *S. salivarius* (Fig. 6) (7). On the other hand, the kinase could also control competence as a muropeptide signaling system. Our experiments with peptidoglycan extracts (Fig. 5C and D) advocate for this possibility as a high concentration of self-muropeptides inhibits competence in an StkP-dependent manner. In line with this, competence in streptococci is transiently activated during the early exponential growth but could not be triggered in stationary phase when the extracellular muropeptide concentration is expected to be high (14, 16, 70). This may suggest that StkP acts as a growth phase sensor to extinct competence at high cell density (Fig. 6).

To conclude, we showed the large potential of combining a genome-wide CRISPRi strategy with multiple screening approaches to connect essential genes involved in physiological pathways to competence development. Besides the well-established ComRS-ComX regulatory pathway, we revealed that disturbance of general functions such as cell envelope assembly, amino acid metabolism, translation, and replication modulates competence activation in *S. salivarius*. This work strengthens our view that competence is a general adaptative response that ensures survival under a broad range of stress conditions. Moreover, the identification of a large set of “competence-associated” genes paves the way to understand novel regulatory cascades interconnecting cell-proliferation impairment and competence activation such as illustrated here for the role of the serine-threonine kinase StkP in cell wall-mediated competence modulation.

## MATERIALS AND METHODS

### Bacterial strains, plasmids, oligonucleotides, and PCR fragments.

Bacterial strains, plasmids, oligonucleotides, and PCR fragments used in this study are listed and described in Tables S1, S2, S3, and S4 in the supplemental material, respectively.

10.1128/msystems.00735-22.4 TABLE S1 List of bacterial strains used in this study. Download Table S1, PDF file, 0.04 MB. Copyright © 2022 Knoops et al. 2022 Knoops et al. https://creativecommons.org/licenses/by/4.0/ This content is distributed under the terms of the Creative Commons Attribution 4.0 International license.

10.1128/msystems.00735-22.5 TABLE S2 List of plasmids used in this study. Download Table S2, PDF file, 0.1 MB. Copyright © 2022 Knoops et al. 2022 Knoops et al. https://creativecommons.org/licenses/by/4.0/ This content is distributed under the terms of the Creative Commons Attribution 4.0 International license.

10.1128/msystems.00735-22.6 TABLE S3 List of oligonucleotides used in this study. Download Table S3, PDF file, 0.01 MB. Copyright © 2022 Knoops et al. 2022 Knoops et al. https://creativecommons.org/licenses/by/4.0/ This content is distributed under the terms of the Creative Commons Attribution 4.0 International license.

10.1128/msystems.00735-22.7 TABLE S4 List of PCR fragments used in this study. Download Table S4, PDF file, 0.04 MB. Copyright © 2022 Knoops et al. 2022 Knoops et al. https://creativecommons.org/licenses/by/4.0/ This content is distributed under the terms of the Creative Commons Attribution 4.0 International license.

### Growth conditions and competence induction.

*S. salivarius* HSISS4 (71) and derivatives were grown at 37°C without shaking in M17 (Difco Laboratories, Detroit, MI) or in chemically defined medium (CDM) (72) supplemented with 1% (wt/vol) glucose (M17G and CDMG, respectively). Chromosomal genetic constructions were introduced in *S. salivarius* via natural transformation (73). We added d-xylose (0.1% to 1% [wt/vol]), IPTG (1 mM), spectinomycin (200 μg/mL), chloramphenicol (5 μg/mL), or erythromycin (10 μg/mL), as required. The synthetic peptides (purity of 95%), were supplied by Peptide 2.0 Inc. (Chantilly, VA) and resuspended first in dimethylformamide (DMF) and diluted in water to reach a low DMF concentration (final concentration of 0.02%). Solid plates inoculated with streptococcal cells were incubated anaerobically (BBL GasPak systems; Becton, Dickinson, Franklin Lakes, NJ) at 37°C.

To induce competence, overnight CDMG precultures were diluted at a final optical density at 600 nm (OD_600_) of 0.05 in 500 μL of fresh CDMG and incubated 100 min at 37°C. Then, the pheromone sXIP (synthetic XIP; LPYFAGCL) and DNA (Gibson assembled PCR products or plasmids) were added and cells were incubated for 3 h at 37°C before plating on M17G agar supplemented with antibiotics when required.

### Library design and construction.

The gRNA library was designed by selecting all the 20-nt sequences followed by a PAM sequence within the genome of HSISS4. Since it was shown that efficient interference in CDSs occurs only with gRNAs targeting the coding strand (34), we filtered the library to keep only gRNAs targeting the coding strand in CDSs (median gRNA number per CDS of 24) and targeting both strands in intergenic regions. We ended up with a high-density library of 83,104 gRNAs resulting in a theoretical base pairing every 25 bp on the HSISS4 genome. Of note, we chose to use a high-density library to target unknown small genetic elements such as small interfering RNA (siRNA) or small peptides. We ordered the gRNAs as single-strand DNA (Twist BioSciences) and amplified the oligonucleotide pool by using the common upstream and downstream region using primers AK475 and AK476. To reduce any amplification sequence bias, we used 10 cycles of amplification.

The PCR products were then purified (Monarch kit; New England Biolabs [NEB]) and Gibson-joined to the preamplified upstream homologous region of the neutral locus *gor* (downstream of *HSISS4_00325*) containing an erythromycin resistance gene and to the downstream homologous region of the same locus fused to a P_3_ constitutive promoter. We performed 20 independent Gibson assemblies, which were later transformed by natural transformation into 20 independent cultures of HSISS4 strains containing at least a lactose-inducible dCas9 module (P_F6_-*lacI*; P*_lac_-dcas9*) (7). Supplemental genetic constructions (P*_xyl2_-comR*, P*_comX_-lacZ*, P*_comX_-luxAB*) were present in those strains depending on the screening strategy. For every library produced, we calculated the number of transformants to obtain at least 15-fold transformants over the diversity rate, ensuring theoretically that 99.9% of the diversity would be present in the library (74). We finally pooled all the transformants in phosphate-buffered saline (PBS; 137 mM NaCl, 2.7 mM KCl, 10 mM Na_2_HPO_4_, 1.8 mM KH_2_PO_4_) and measured OD_600_ prior to storage at −80°C.

### CRISPRi transformation screen.

For the spontaneous transformation screen, we first introduced the gRNA library in the lactose-inducible strain harboring a P*_comX_* luciferase reporter system (P_F6_*-lacI*, P*_lac_-dcas9*, P*_comX_-luxAB*). We next diluted the cells in 15 mL of fresh CDMG supplemented with 1 mM IPTG at an OD_600_ of 0.01. We grew this culture at 37°C for 8 h and added every 30 min a PCR-amplified product consisting of a chloramphenicol cassette with 2,000-bp up and down homologous recombination arms at a final concentration of 0.25 nM. We centrifuged this culture, plated it on chloramphenicol plates, and grew it overnight. Colonies were picked, and donor DNA integration was confirmed by PCR. We next amplified the locus containing the gRNAs before Sanger sequencing.

### CRISPRi gRNA depletion screen.

For the gRNA depletion analysis, we used the same strain as described above. After introducing the gRNAs in this background, we spread the resulting library onto three different solid media (M17G, M17G with 100 μM IPTG, and M17G with 100 μM IPTG and 1% xylose), resulting in an average of 9.6 × 10^6^ CFU per large plate. Technical replicates (*n *= 4) were incubated 16 h at 37°C to yield an estimated mean of ~12 generations. Cells were then collected, pooled in PBS buffer, and homogenized for each replicate. After genomic extraction (GenElute; Sigma-Aldrich) from at least 1.5 × 10^9^ CFU per replicate, we PCR amplified the locus containing the gRNAs prior to their deep sequencing. We used an optimized PCR protocol with a high primer concentration (5 μM), a low level of template genomic DNA (2 ng/μL), and a low number of cycles (15 cycles) to avoid any chimeric products due to the highly randomized gRNA sequences. The 219-bp amplicons were next gel purified (Monarch DNA gel extraction kit; NEB) and sent with a minimum amount of 4 pmol for Illumina sequencing (Genewiz). High-Seq Illumina sequencing was performed with 30% PhiX and generated an average of 30 million reads per replicate.

### CRISPRi β-galactosidase activity screen.

We first produced a new genetic background by introducing into the strain described above an ectopic copy of *comR* under the control of a xylose-inducible promoter (P*_xyl2_-comR*) together with a chloramphenicol resistance cassette at the neutral locus *suc* (upstream of *HSISS4_01641*). We next fused the promoter of *comX* to the native *lacZ* gene (P*_comX_-lacZ*) together with a spectinomycin resistance cassette and introduced the gRNA library into this strain. The resulting library was spread on M17 with 0.5% glucose and 0.5% lactose (M17GL), 100 μM IPTG, 1% xylose, and 100 mg/mL X-Gal for screening dark blue (highly competent) and white (competence loss) colonies. A total of 158 dark blue and 155 white clones from the screening of ~94,000 colonies were reisolated for phenotype confirmation. Luciferase tests (P*_comX_-luxAB*) were performed in comparison with the parental strain harboring no gRNA. Clones with the most dissimilar luciferase phenotypes (141 dark blue and 68 white clones) were selected, and gRNAs were amplified by PCR for Sanger sequencing.

### NGS analysis.

We used the MAGeCK algorithm to map the reads on the HSISS4 genome (41). Approximately 30% of total reads were mapped, producing about 10 million reads per replicate. Following the MAGeCK guidelines, we next pooled the reads from the 4 replicates, ultimately generating a total of 40 million reads per condition. In a first analysis, we compared the gRNA depletion under the IPTG-induced condition with the mock to determine all the essential genes from strain HSISS4. For the sake of clarity, we compared only gRNAs targeting CDSs, since gRNAs targeting intergenic regions are much more complicated to determine. We next compared the depletion of gRNAs for each gene under the IPTG- and IPTG-xylose-induced conditions to that under the mock condition by using the MAGeCK algorithm. The depletion scores generated per gene for the two induced conditions were then plotted against each other, a linear regression was fitted to the plot (*lm* function, R package), and outliers were identified by standardizing the residuals. We also compared directly the IPTG- and IPTG-plus-xylose-induced conditions (i.e., Ci versus Ci+C) with the MAGeCK algorithm to generate statistical data by robust rank aggregation (RRA) to confirm the identification of outliers.

### COG analysis.

The conserved domain database of NCBI was used to infer functions of the genes from the genome of HSISS4 (44, 75), and only the highest-scoring function for each gene was retained. The number of genes of the whole genome involved in each function prediction was then calculated, generating a function prediction frequency matrix. This matrix was then used to weight the number of genes with a specific predicted function highlighted in the different screens.

### Luciferase assay.

Overnight precultures were diluted at a final OD_600_ of 0.05. A volume of 300 μL of culture was transferred in the wells of a sterile covered white microplate with a transparent bottom (Greiner, Alphen a/d Rijn, The Netherlands). These culture samples were supplemented with d-xylose, IPTG, or peptidoglycan extracts if stated. Growth (OD_600_) and luciferase (Lux) activity (expressed in relative light units [RLU]) were monitored at 10-min intervals during 8 to 24 h in a Hidex Sense microplate reader (Hidex, Lemminkäisenkatu, Finland). Specific Lux activity was obtained by dividing Lux activity by the OD_600_ and summing all the data obtained over time. When stated, biological or technical triplicates were averaged. Statistical analyses of simple and multiple comparisons to the control mean were performed with *t* test (unilateral distribution, heteroscedastic) and one-way analysis of variance (ANOVA) with Dunnett’s test, respectively. For both, standard deviations and *P* values were calculated.

### Transformation test.

The CDMG preculture of HSISS4 and derivatives was diluted in 500 μL of CDMG supplemented with 1 mM IPTG at an OD_600_ of 0.005. The culture was grown at 37°C for 8 h, and we added every 30 min a PCR-amplified product consisting of a chloramphenicol resistance cassette surrounded by up and down homologous recombination arms (2,000 bp each) at a final concentration of 0.25 nM. We next performed serial dilution of the culture and spread the various dilutions on M17G plates supplemented with or without 5 μg/mL chloramphenicol. We next calculated the transformation rate based on the CFU numbers of the two plates.

### Peptidoglycan extracts.

Peptidoglycan extracts were prepared as previously reported (55). Cultures of 100 mL of *S. salivarius* HSISS4 or B. subtilis 168 were grown to an OD_600_ of ~1.2 in M17 or LB medium, respectively. Cells were collected by centrifugation, washed with 0.8% NaCl, resuspended in hot 4% SDS, boiled for 30 min, and incubated at room temperature overnight. The suspension was then boiled for 10 min, and the SDS-insoluble cell wall material was collected by centrifugation at 12,000 × *g* for 15 min at room temperature. The pellet containing cell wall peptidoglycan was washed four times with water and finally resuspended in 1 mL sterile water. The resuspended peptidoglycan was next digested with mutanolysin (10 μg/mL) overnight at 37°C prior to inactivation of mutanolysin at 80°C for 20 min.

### Data availability.

All gRNA sequencing data were deposited in the GEO database under accession number GSE204976 (https://www.ncbi.nlm.nih.gov/geo/query/acc.cgi?acc=GSE204976). All other data supporting the findings of this study are available in the article or in the supplemental material.
