# Supplementary figures and images for: A Genome-Wide CRISPR Interference Screen Reveals an StkP-Mediated Connection between Cell Wall Integrity and Competence in Streptococcus salivarius
Source: mSystems. 2022 Nov 7;7(6):e00735-22. doi: 10.1128/msystems.00735-22 (PMC9765292; doi:10.1128/msystems.00735-22)

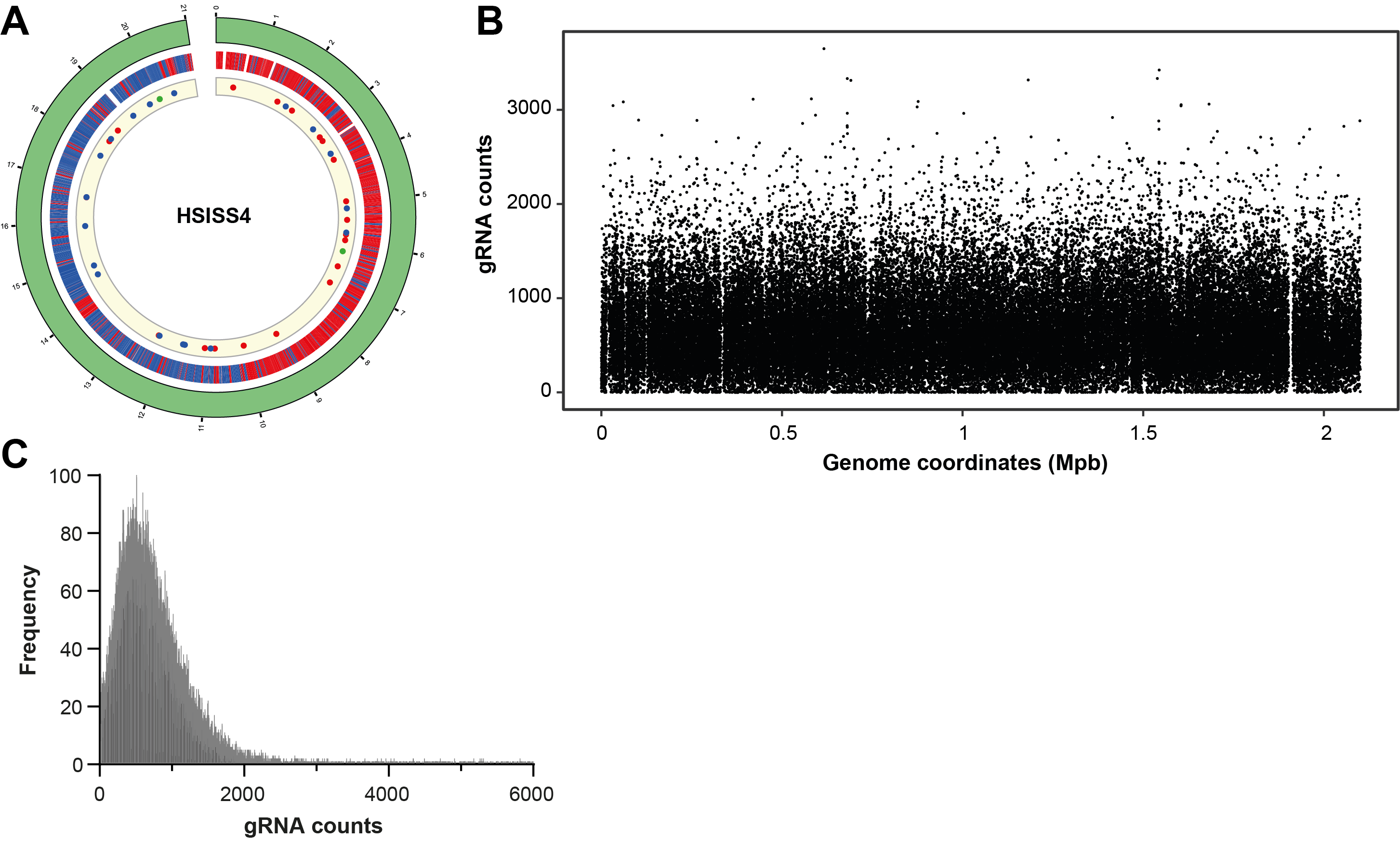

Supplement: FIG S1 [file msystems.00735-22-s0001.tif]

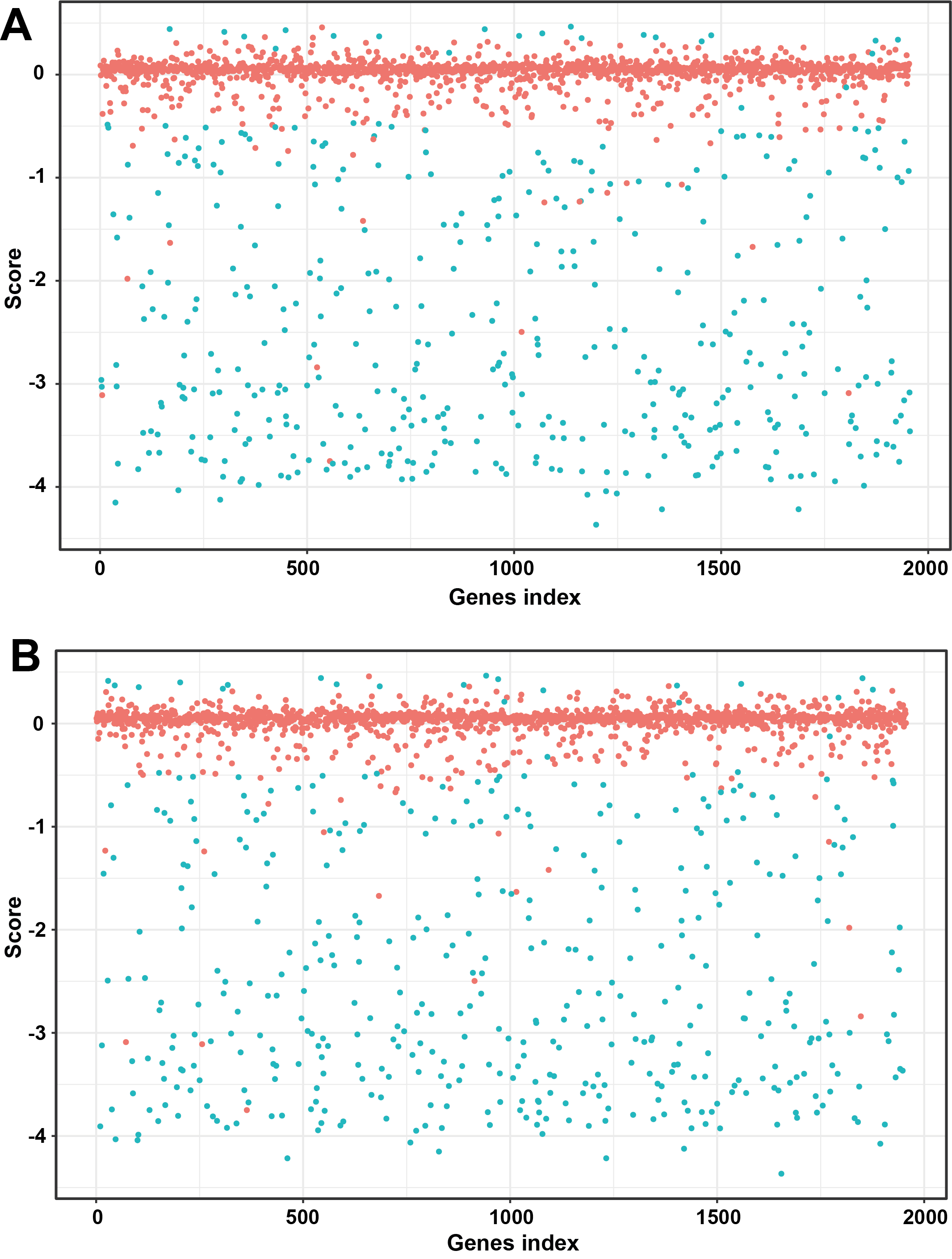

Supplement: FIG S2 [file msystems.00735-22-s0002.tif]

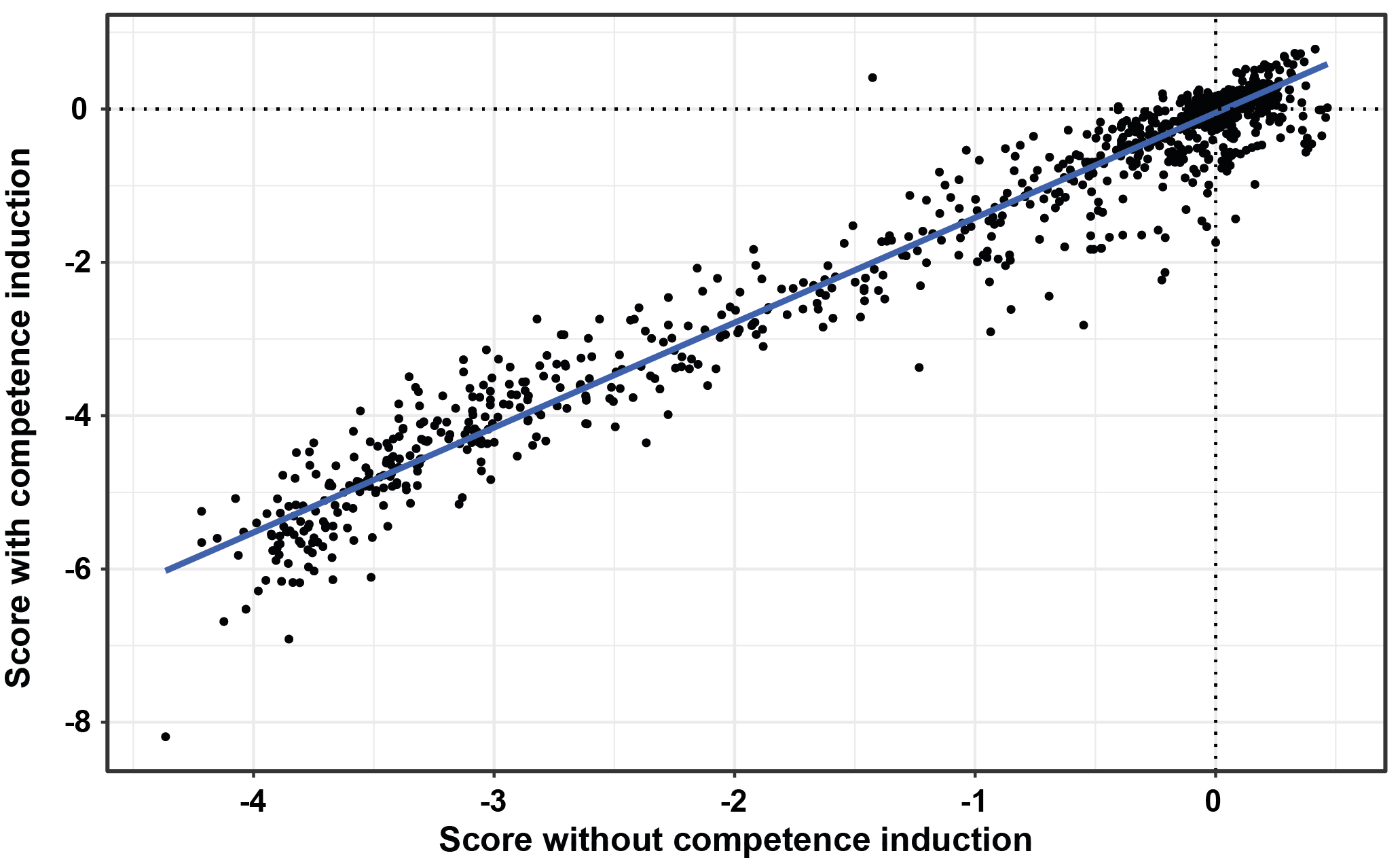

Supplement: FIG S3 [file msystems.00735-22-s0003.tif]
