# Supplementary material for: A Genome-Wide CRISPR Interference Screen Reveals an StkP-Mediated Connection between Cell Wall Integrity and Competence in Streptococcus salivarius
Source: mSystems. 2022 Nov 7;7(6):e00735-22. doi: 10.1128/msystems.00735-22 (PMC9765292; doi:10.1128/msystems.00735-22)
Supplement: TABLE S1 [file msystems.00735-22-s0004.pdf]

**Table S1. List of bacterial strains used in this study**

| Strain | Characteristics | Reference/source |
|---|---|---|
| ***Streptococcus salivarius*** | | |
| HSISS4 | Wild-type gastro-intestinal tract isolate | (1) |
| AK0004 | HSISS4 *tRNA*$_{thr}$::P$_{comX}$-*luxAB-spec*<br>*tRNA*$_{ser}$::P$_{xyl2}$-*comR-cat* | (2) |
| AK0033 | HSISS4 *tRNA*$_{thr}$::P$_{F6}$-*lacI-lox72*<br>*tRNA*$_{ser}$::P$_{lac}$-*dcas9-lox72*<br>*tnpII::P*$_{comX}$-*luxAB-lox72* | (2) |
| AK0034 | HSISS4 *tRNA*$_{thr}$::P$_{F6}$-*lacI-lox72*<br>*tRNA*$_{ser}$::P$_{lac}$-*dcas9-lox72*<br>*tnpII::P*$_{comR}$-*luxAB-lox72* | (2) |
| AK0046 | HSISS4 *tRNA*$_{thr}$::P$_{F6}$-*lacI-lox72*<br>*tRNA*$_{ser}$::P$_{lac}$-*dcas9-lox72*<br>*tnpII::P*$_{comX}$-*luxAB-lox72*<br>*SUC::P*$_{xyl2}$-*comR-lox72* | (3) |
| AK0065 | HSISS4 *tRNA*$_{thr}$::P$_{F6}$-*lacI-lox72*<br>*tRNA*$_{ser}$::P$_{lac}$-*dcas9-lox72*<br>*tnpII::P*$_{comX}$-*luxAB-lox72*<br>*SUC::P*$_{xyl2}$-*comR-cat*<br>*P*$_{comX}$-*lacZ-spc* | This work |
| AK0066 | HSISS4 *tRNA*$_{thr}$::P$_{F6}$-*lacI-lox72*<br>*tRNA*$_{ser}$::P$_{lac}$-*dcas9-lox72*<br>*tnpII::P*$_{comX}$-*luxAB-lox72*<br>*SUC::P*$_{xyl2}$-*comR-lox72*<br>*GOR::P*$_{3}$-*g_30(HSISS4_01622)-erm* | This work |
| AK0067 | HSISS4 *tRNA*$_{thr}$::P$_{F6}$-*lacI-lox72*<br>*tRNA*$_{ser}$::P$_{lac}$-*dcas9-lox72*<br>*tnpII::P*$_{comX}$-*luxAB-lox72*<br>*SUC::P*$_{xyl2}$-*comR-lox72*<br>*GOR::P*$_{3}$-*g_27(HSISS4_01391)-erm* | This work |
| AK0068 | HSISS4 *tRNA*$_{thr}$::P$_{F6}$-*lacI-lox72*<br>*tRNA*$_{ser}$::P$_{lac}$-*dcas9-lox72*<br>*tnpII::P*$_{comX}$-*luxAB-lox72*<br>*SUC::P*$_{xyl2}$-*comR-lox72*<br>*GOR::P*$_{3}$-*g_31(HSISS4_00663)-erm* | This work |
| AK0069 | HSISS4 *tRNA*$_{thr}$::P$_{F6}$-*lacI-lox72*<br>*tRNA*$_{ser}$::P$_{lac}$-*dcas9-lox72*<br>*tnpII::P*$_{comX}$-*luxAB-lox72*<br>*SUC::P*$_{xyl2}$-*comR-lox72*<br>*GOR::P*$_{3}$-*g_32(HSISS4_00805)-erm* | This work |
| AK0070 | HSISS4 *tRNA*$_{thr}$::P$_{F6}$-*lacI-lox72*<br>*tRNA*$_{ser}$::P$_{lac}$-*dcas9-lox72*<br>*tnpII::P*$_{comX}$-*luxAB-lox72* | This work |

| | | |
|---|---|---|
| | *SUC*::$P_{xyl2}$-*comR-lox72*<br>*GOR*::$P_3$-*g_35(HSISS4_01302)-erm* | |
| AK0071 | HSISS4 $tRNA_{thr}$::$P_{F6}$-*lacI-lox72*<br>$tRNA_{ser}$::$P_{lac}$-*dcas9-lox72*<br>*tnpII*::$P_{comX}$-*luxAB-lox72*<br>*SUC*::$P_{xyl2}$-*comR-lox72*<br>*GOR*::$P_3$-*g_26(gpmB-dacB-mur3)-erm* | This work |
| AK0072 | HSISS4 $tRNA_{thr}$::$P_{F6}$-*lacI-lox72*<br>$tRNA_{ser}$::$P_{lac}$-*dcas9-lox72*<br>*tnpII*::$P_{comX}$-*luxAB-lox72*<br>*SUC*::$P_{xyl2}$-*comR-lox72*<br>*GOR*::$P_3$-*g_37(clpC)-erm* | This work |
| AK0073 | HSISS4 $tRNA_{thr}$::$P_{F6}$-*lacI-lox72*<br>$tRNA_{ser}$::$P_{lac}$-*dcas9-lox72*<br>*tnpII*::$P_{comX}$-*luxAB-lox72*<br>*SUC*::$P_{xyl2}$-*comR-lox72*<br>*GOR*::$P_3$-*g_38(clpC)-erm* | This work |
| AK0074 | HSISS4 $tRNA_{thr}$::$P_{F6}$-*lacI-lox72*<br>$tRNA_{ser}$::$P_{lac}$-*dcas9-lox72*<br>*tnpII*::$P_{comX}$-*luxAB-lox72*<br>*SUC*::$P_{xyl2}$-*comR-lox72*<br>*GOR*::$P_3$-*g_39(pepF)-erm* | This work |
| AK0075 | HSISS4 $tRNA_{thr}$::$P_{F6}$-*lacI-lox72*<br>$tRNA_{ser}$::$P_{lac}$-*dcas9-lox72*<br>*tnpII*::$P_{comX}$-*luxAB-lox72*<br>*SUC*::$P_{xyl2}$-*comR-lox72*<br>*GOR*::$P_3$-*g_40(scuR/sarF)-erm* | This work |
| AK0076 | HSISS4 $tRNA_{thr}$::$P_{F6}$-*lacI-lox72*<br>$tRNA_{ser}$::$P_{lac}$-*dcas9-lox72*<br>*tnpII*::$P_{comX}$-*luxAB-lox72*<br>*SUC*::$P_{xyl2}$-*comR-lox72*<br>*GOR::P3-g_41(pepXP)-erm* | This work |
| AK0077 | HSISS4 $tRNA_{thr}$::$P_{F6}$-*lacI-lox72*<br>$tRNA_{ser}$::$P_{lac}$-*dcas9-lox72*<br>*tnpII*::$P_{comX}$-*luxAB-lox72*<br>*SUC*::$P_{xyl2}$-*comR-lox72*<br>*GOR*::$P_3$-*g_42(carB)-erm* | This work |
| AK0078 | HSISS4 $tRNA_{thr}$::$P_{F6}$-*lacI-lox72*<br>$tRNA_{ser}$::$P_{lac}$-*dcas9-lox72*<br>*tnpII*::$P_{comX}$-*luxAB-lox72*<br>*SUC*::$P_{xyl2}$-*comR-lox72*<br>*GOR*::$P_3$-*g_43(IG664598)-erm* | This work |
| AK0079 | HSISS4 $tRNA_{thr}$::$P_{F6}$-*lacI-lox72*<br>$tRNA_{ser}$::$P_{lac}$-*dcas9-lox72* | This work |

| | | |
|---|---|---|
| | *tnpII::*$P_{comR}$*-luxAB-lox72*<br>*GOR::*$P_3$*-g_23(stkP)-erm* | |
| AK0080 | HSISS4 *tRNA*$_{thr}$*::*$P_{F6}$*-lacI-lox72*<br>*tRNA*$_{ser}$*::*$P_{lac}$*-dcas9-lox72*<br>*tnpII::*$P_{comX}$*-luxAB-lox72*<br>*SUC::*$P_{xyl2}$*-comR-lox72*<br>*GOR::*$P_3$*-g_23(stkP)-erm* | This work |
| ***Bacillus subtilis*** | | |
| 168 | Wild-type | J. Mahillon, Laboratory collection |

## REFERENCES


1. Van Den Bogert B, Boekhorst J, Herrmann R, Smid EJ, Zoetendal EG, Kleerebezem M. 2013. Comparative genomics analysis of Streptococcus isolates from the human small intestine reveals their adaptation to a highly dynamic ecosystem. *PLoS One* 8:83418.

2. Knoops A, Vande Capelle F, Fontaine L, Verhaegen M, Mignolet J, Goffin P, Mahillon J, Sass A, Coenye T, Ledesma-García L, Hols P. 2022. The CovRS Environmental Sensor Directly Controls the ComRS Signaling System To Orchestrate Competence Bimodality in Salivarius Streptococci. MBio https://doi.org/10.1128/mbio.03125-21.

3. Knoops A, Ledesma-García L, Waegemans A, Lamontagne M, Decat B, Degand H, Morsomme P, Soumillion P, Delvigne F, Hols P. 2022. Competence shut-off by intracellular pheromone degradation in salivarius streptococci. *PLOS Genet* 18:e1010198.
