# Supplementary material for: A Genome-Wide CRISPR Interference Screen Reveals an StkP-Mediated Connection between Cell Wall Integrity and Competence in Streptococcus salivarius
Source: mSystems. 2022 Nov 7;7(6):e00735-22. doi: 10.1128/msystems.00735-22 (PMC9765292; doi:10.1128/msystems.00735-22)
Supplement: TABLE S2 [file msystems.00735-22-s0005.pdf]

Table S2. List of plasmids used in this study

Plasmid	Characteristic(s)	Reference
pGhost-cre	Thermosensitive replication origin vector in *S. salivarius*, encoding the Cre recombinase; ery^R	(1)
pGIUD0855ery	pUC18 derivative containing the *erm* gene	(1)
pJUD*specmut1*-*gfp*+ter	Terminator associated-*gfp*+ ORF cloned in cloned in pJUDspecmut1	(2)
pJIM*cat*	pJIM4900 derivative with a *cat* cassette	(2)

REFERENCES

1. Fontaine L, Dandoy D, Boutry C, Delplace B, de Frahan MH, Fremaux C, Horvath P, Boyaval P, Hols P. 2010. Development of a versatile procedure based on natural transformation for marker-free targeted genetic modification in *Streptococcus thermophilus*. *Appl Environ Microbiol* 76:7870–7.

2. Mignolet J, Fontaine L, Sass A, Nannan C, Mahillon J, Coenye T, Hols P. 2018. Circuitry Rewiring Directly Couples Competence to Predation in the Gut Dweller *Streptococcus salivarius*. *Cell Rep* 22:1627–1638.
