# Supplementary material for: A Genome-Wide CRISPR Interference Screen Reveals an StkP-Mediated Connection between Cell Wall Integrity and Competence in Streptococcus salivarius
Source: mSystems. 2022 Nov 7;7(6):e00735-22. doi: 10.1128/msystems.00735-22 (PMC9765292; doi:10.1128/msystems.00735-22)
Supplement: TABLE S3 [file msystems.00735-22-s0006.pdf]

Table S3. List of oligonucleotides used in this study

Name	Sequence (5' to 3')
**AK396**	TCAACCTCCTATTAATAGATATAATTTTTG
**AK458**	GTAGCAACACTCTTGTTTAAAG
**AK459**	GTGGCTGAATTATCAAAATAAATC
**AK465**	TATAGATTTCATTGCTGGAC
**AK484**	GCCTTAGCCAAATCGTAATC
**AK485**	AACAGGAGGTTTTCGTAATGG
**AK518**	GTATTCAAAAAACAACTAACAGG
**AK519**	CCAACGTCCAGCAATGAAATCTATATAAGGAAGATAAATCCCATAAGG
**AK520**	GTTATTACCTTCAAAAAAGGAGAATAATCTTTCACGTTACTAAAGGGAATGTA
**Dn.Rv.lox71**	TTCACGTTACTAAAGGGAATGTA
**ML7**	GGAGGCAAAGTCCAATAATACTTAAGGAAGATAAATCCCATAAG
**ML8**	GTTATTACCTTCAAAAAAGGAGAATAATCTTTCACGTTACTAAAGGGAATG
**ML9**	AGATTATTCTCCTTTTTTGAAG
**ML11**	GTTTGAATTTTTTCAGTCGTGTTCATTCAACCTCCTATTAATAGATATAATTTTTG
**ML13**	TTAGTTGAGTGGTTCAATCATG
**Up.Fw.lox66**	TAAGGAAGATAAATCCCATAAGG
