# Supplementary material for: A Genome-Wide CRISPR Interference Screen Reveals an StkP-Mediated Connection between Cell Wall Integrity and Competence in Streptococcus salivarius
Source: mSystems. 2022 Nov 7;7(6):e00735-22. doi: 10.1128/msystems.00735-22 (PMC9765292; doi:10.1128/msystems.00735-22)
Supplement: TABLE S4 [file msystems.00735-22-s0007.pdf]

Table S4. List of PCR fragments used in this study

Strain/DNA	PCR fragment	Template DNA	Primer 1	Primer 2
AK0065	Up HR of *lacZ* locus	HSISS4	ML13	ML11
	Promoter of *comX*, P_{comX}	HSISS4	AK396	AK465
	Spectinomycin resistance cassette, *spc*	pJUD*specmut1-gfp*+ter	AK519	AK520
	Dw HR of *lacZ* locus	HSISS4	ML9	AK518
AK0066	P_3-g_30(*HSISS4_01622*) fused to the two HR of the *GOR* locus together with *erm*	Selected colony from transformation screen #2	AK458	AK459
AK0067	P_3-g_27(*HSISS4_01391*) fused to the two HR of the GOR locus together with *erm*	Selected colony from transformation screen #5	AK458	AK459
AK0068	P_3-g_31(*HSISS4_00663*) fused to the two HR of the GOR locus together with *erm*	Selected colony from transformation screen #6	AK458	AK459
AK0069	P_3-g_32(*HSISS4_00805*) fused to the two HR of the GOR locus together with *erm*	Selected colony from transformation screen #9	AK458	AK459
AK0070	P_3-g_35(*HSISS4_01302*) fused to the two HR of the GOR locus together with *erm*	Selected colony from transformation screen #13	AK458	AK459
AK0071	P_3-g_26(*gpmB-dacB-mur3*) fused to the two HR of the GOR locus together with *erm*	Selected colony from transformation screen #16	AK458	AK459
AK0072	P_3-g_37(*clpC*) fused to the two HR of the GOR locus together with *erm*	Selected colony from transformation screen #17	AK458	AK459
AK0073	P_3-g_38(*clpC*) fused to the two HR of the GOR locus together with *erm*	Selected colony from transformation screen #18	AK458	AK459
AK0074	P_3-g_39(*pepF*) fused to the two HR of the GOR locus together with *erm*	Selected colony from transformation screen #21	AK458	AK459
AK0075	P_3-g_40(*scuR/sarF*) fused to the two HR of the GOR locus together with *erm*	Selected colony from transformation screen #22	AK458	AK459
AK0076	P_3-g_41(*pepXP*) fused to the two HR of the GOR locus together with *erm*	Selected colony from transformation screen #23	AK458	AK459
AK0077	P_3-g_42(*carB*) fused to the two HR of the GOR locus together with *erm*	Selected colony from transformation screen #25	AK458	AK459
AK0078	P_3-g_43(*IG664598*) fused to the two HR of the GOR locus together with *erm*	Selected colony from transformation screen #28	AK458	AK459
AK0079, AK0080	P_3-g_27(*stkP*) fused to the two HR of the GOR locus together with *erm*	Selected colony from the *lacZ* screen	AK458	AK459
Δ*lacZ::cat*	Up HR of *lacZ* locus (2000 bp)	HSISS4	AK484	ML7
	Chloramphenicol resistance cassette, *cat*	pJIM*cat*	Up.Fw.lox66	Dn.Rv.lox71
	Dw HR of *lacZ* locus (2000 bp)	HSISS4	ML8	AK485
